# A New Antitumor Direction: Tumor-Specific Endothelial Cells

**DOI:** 10.3389/fonc.2021.756334

**Published:** 2021-12-20

**Authors:** Jing Liang, Shouqi Wang, Guowei Zhang, Baoyu He, Qingli Bie, Bin Zhang

**Affiliations:** ^1^ Department of Laboratory Medicine, Affiliated Hospital of Jining Medical University, Jining Medical University, Jining, China; ^2^ Department of Gastrointestinal Surgery, Affiliated Hospital of Jining Medical University, Jining Medical University, Jining, China; ^3^ Institute of Forensic Medicine and Laboratory Medicine, Jining Medical University, Jining, China

**Keywords:** tumor-specific endothelial cells, tumor heterogeneity, tumor angiogenesis, tumor microenvironment, antiangiogenic therapy

## Abstract

Targeting tumor blood vessels is an important strategy for tumor therapies. At present, antiangiogenic drugs are known to have significant clinical effects, but severe drug resistance and side effects also occur. Therefore, new specific targets for tumor and new treatment methods must be developed. Tumor-specific endothelial cells (TECs) are the main targets of antiangiogenic therapy. This review summarizes the differences between TECs and normal endothelial cells, assesses the heterogeneity of TECs, compares tumorigenesis and development between TECs and normal endothelial cells, and explains the interaction between TECs and the tumor microenvironment. A full and in-depth understanding of TECs may provide new insights for specific antitumor angiogenesis therapies.

## 1 Introduction

Tumor angiogenesis refers to the formation of new blood vessels in tumors.Tumor blood vessels provide oxygen and nutrients for tumor growth, remove waste from tumor tissues, and provide pathways for tumor metastasis.If a solid tumor has insufficient amounts of blood vessels, then it can only grow to a critical size of 1–2 mm (or approximately 10^6^ cells) (1). In recent years, many studies have identified significant differences in the structure and function between tumor vasculature and normal vasculature. Normal blood vessels have regular hierarchical structures that are responsible for blood flow throughout the body and maintain normal physiological activities, and these structures include arteries, veins and capillaries. Tumor blood vessels, however, are highly irregular in shape, and they are swollen and twisting and have many blind ends, which results in abnormal vascular function, including increased vascular permeability, leakage and bleeding, and blood flow disorder (2, 3).

The specificity of tumor-specific endothelial cells (TECs) is one of the main reasons for tumor vascular anomalies. Blood vessels are composed of ECs and pericytes, which are responsible for the contraction and relaxation of blood vessels. In normal blood vessels, ECs are mostly in a static state. In tumors, hypoxia and chronic growth factor stimulation may cause endothelial dysfunction (4). Increasing evidence has shown that these abnormalities can lead to the development of cancer. Initial hypotheses suggested that TECs were genetically stable normal somatic cells that were not prone to mutation and drug resistance and remained consistent in various tumors, which would allow multiple types of cancer to be treated by a single antiangiogenic drug (5). However, in recent years, many researchers have found that TECs are not ordinary somatic cells but rather are heterogeneous in many aspects relative to normal endothelial cells (NECs), which contradicts previous assumptions (6, 7). Therefore, the study of TECs will provide targets or directions for tumor therapy. Because most current antiangiogenic drugs are nonspecific, they cause damage to NECs, thus leading to fatal side effects, such as intestinal perforation and bleeding in the later stages (8, 9).Therefore, exploring specific targets for TECs have potential for antitumor therapy.

The tumor microenvironment (TME) is essential for tumor progression, which accelerates metastasis and increases tumor malignancy. TME refers to the local environment for tumor survival. A large number of tumor cells infiltrate in TME, includes stromal cells, TECs and immune cells (10). TME provides an acidic and hypoxic environment containing a large number of cytokines (11). TME plays an important role in the process that TECs promote tumor progression and drug resistance (12). Among them, tumor-associated macrophages (TAMs) and tumor-associated fibroblasts (CAFs) have been found to promote the proliferation, migration and tube formation of HUVECs (13–15). And Myeloid-Derived Suppressor Cells (MDSCs) and extracellular matrix (ECM) alsoregulate the function of ECs (16, 17). Therefore, to study the interaction between TECs and TME is necessary to target for TECs therapy.

## 2 Antitumor Angiogenesis Therapy

Fifty years ago, Judah Folkman first emphasized that angiogenesis was an important process for the growth and proliferation of solid tumors (18) and proposed that antiangiogenesis may be a potential method for treatment various cancers. In recent years, many factors and related receptors that promote angiogenesis have been confirmed, including vascular endothelial growth factor (VEGF) (19), platelet-derived growth factor (PDGF) (20, 21) and angiopoietin (Ang) (22), and antitumor angiogenesis drugs have been developed.

**Figure 1:**
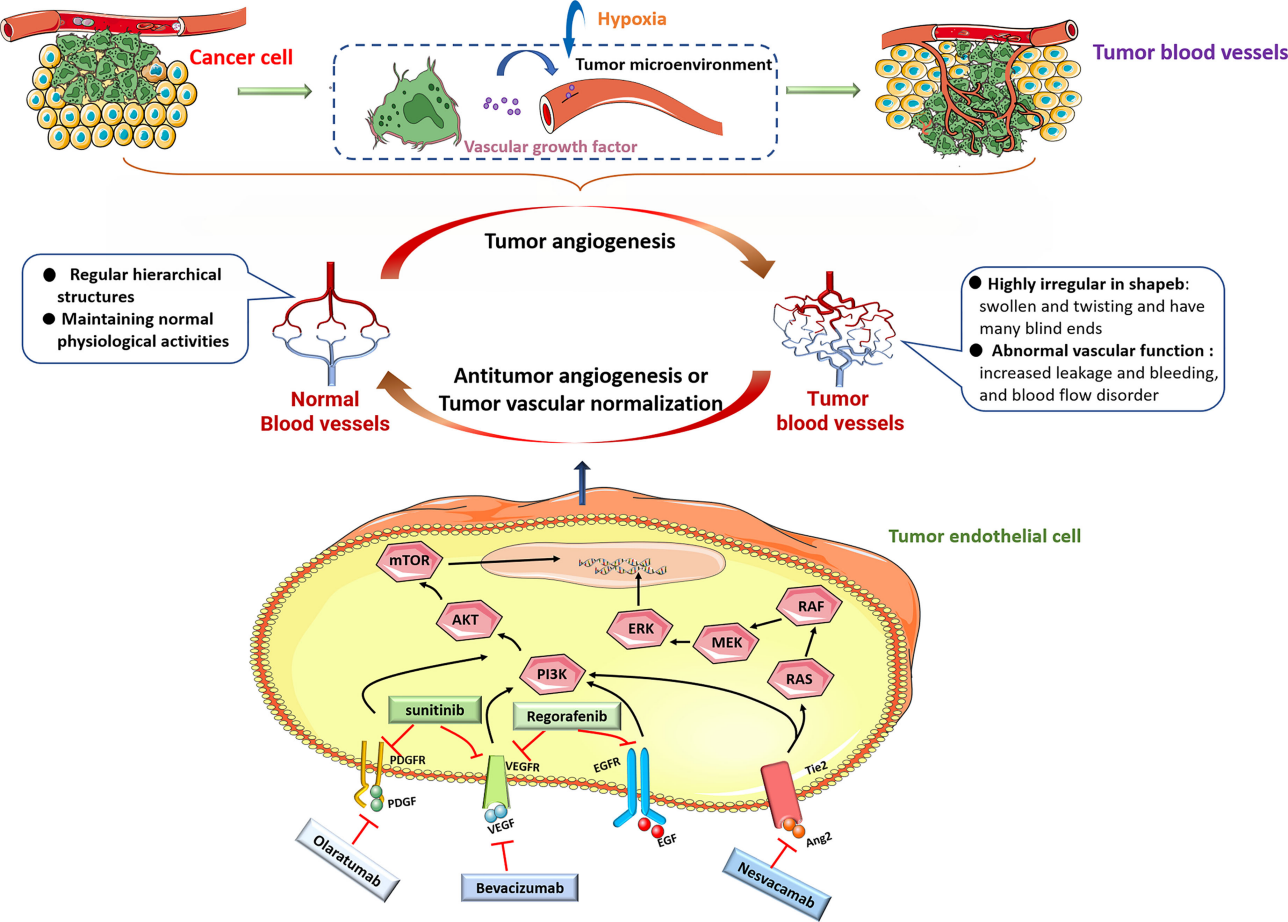
Under pathological conditions including hypoxia, most tumor cells upregulate the expression of many angiogenic factors, including VEGF, PDGF, EGF and Ang, and secrete them into the tumor microenvironment. When these molecules bind to their cognate receptors, then stimulate downstream signaling, including PI3K/AKT and RAS/MEK/ERK signaling pathways to promote tumor growth and angiogenesis. Monoclonal antibodies beracizumab, olaratumab, nesvacamab and small molecule drugs sunitinib and regorafenib can block the interaction between pro-angiogenic factors and their receptors, and inhibit tumor angiogenesis.

VEGF is considered a key factor in inducing tumor angiogenesis and is research hotspot as a key target for antitumor vascular therapy. VEGF activates MAPK, PI3K and other signals in ECs by binding to VEGF receptors (VEGFR1-3) to promote the formation of new blood vessels, increase vascular permeability, and regulate tumor angiogenesis (23–25). In recent decades, VEGF has been used as a therapeutic target to inhibit angiogenesis and promote the normalization of tumor blood vessels, and has achieved great success. Bevacizumab is the first approved antitumor angiogenesis therapy monoclonal antibody, and it can specifically bind to VEGF to inhibit tumor angiogenesis (26). But, the effect of bevacizumab treatment alone is limited. Minjian et al. observed that a significant upregulation of VEGF and downregulation of β-FGF and ANG1 in colon cancer-derived endothelial cells treated with bevacizumab alone, which might activate a potential self-regulating mechanism of angiogenic growth factors and also explained why current antiangiogenic therapy with bevacizumab alone has limited effects in prolonging the survival of colon cancer patients (27). Clinical studies have confirmed that a combination of bevacizumab and chemotherapy drugs can significantly prolong the survival period of tumor patients and achieve antitumor effects (28–30). Nadine et al. found that telomerase regulates VEGF expression and secretion through its catalytic subunit hTERT in gastrointestinal cancer cells, and VEGF inhibition with bevacizumab increased hTERT expression which further increased VEGFR1 and VEGFR2 expression. They suggested the combination of bevacizumab with telomerase inhibitors could improve tumor cell response to anti-VEGF treatment (31). The VEGF pathway coordinates with many other signaling pathways, such as Ang/Tie receptor and PDGF/PDGFR signaling targeted by specific inhibitors nesvacumab and olaratumab, participates in tumor angiogenesis. Tyrosine kinase inhibitors (TKIs) are small molecule drugs that can inhibit the kinase activity of different receptors and their downstream signal transduction. Several studies revealed sunitinib, a TKI, not only targets VEGFR (32) but also inhibits PDGFR (33) and FGFR (34).Sunitinib has been used to treat a variety of cancers. Similarly, studies have reported that sunitinib treatment alone can cause ECs senescence, loose ECs connections, and promote tumor cell migration through the endothelial barrier (35). Based on the above research reports, treatment with one antitumor angiogenesis drug alone have great limit effects on cancer therapy (**Figure 1**).

Previously, antiangiogenic drugs were thought to be less toxic than other cytotoxic drugs; however, they subsequently been found have serious side effects (hypertension (36), bleeding (37),gastrointestinal perforation (8), etc.), with vascular toxicity particularly prominent. Theseside effects are associated with the inhibitory role of most current antiangiogenic drugs on cell signaling pathways, such as VEGF/VGEFR, which has a negative impact on the survival of NECs (38).Antiangiogenic drugs and their side effects are shown in (**Table 1**) (39–49). An important goal of cancer treatment is to develop new and safer tumor-specific antiangiogenic drugs.

**Table 1:** Anti-angiogenic drugs.

Type	Drug	Target	Manufacturer	Approval	Indication	Side Effects
Monoclonal antibodies	Bevacizumab	VEGF	Genentech	2004	Colorectal cancer, Lung cancer, Cervical cancer, Glioblastoma, Ovarian cancer	Fatigue, pain, headache, abdominal pain, constipation, diarrhea, nausea, vomiting, anorexia, hemorrhage, dyspnea, Hypertension
	Cetuximab	EGFR	ImClone	2004	Colorectal cancer, Head and neck cancers	Fatigue, weakness, pain, headache, insomnia, weight loss, skin toxicities, GI toxicities, cough, dyspnea, fever, pharyngitis
	Panitumumab(Vectibix)	EGFR	Amgen	2005	Colorectal cancer	Fatigue, ocular toxicity, nausea, diarrhea, vomiting, skin toxicity, dyspnea reactions
	Ramucirumab	VEGFR2	Imclone	2014	Colorectal cancer, Lung cancer, Gastric cancer,	Hypertension, diarrhea
	Necitumumab	EGFR	Eli Lilly	2015	Lung cancer	Acne, diarrhea, vomiting, mouth sores, vision changes, tearing or itching, red and swollen nails, itching
	Olaratumab	PDGFR	Eli Lilly	2016	Sarcoma	Nausea, fatigue, musculoskeletal pain, mucositis, hair loss, vomiting, diarrhea, loss of appetite, abdominal pain, neuropathy, headache
Tyrosine kinase inhibitors	Imatinib	PDGFR, SCFR	Novartis Abl	2001	Chronic myelocytic leukemia, Gastrointestinal Stromal Tumors	Difficulty breathing, rapid heartbeat, insomnia, coughing up blood or pink mucus, chest pain, frequent urination, fever, jaundice, bloody stools, skin bruises, fatigue
	Gefitinib	EGFR	AstraZeneca	2003	Nonsmall-cell lung cancer	Diarrhea, rash, itching, dry skin, acne
	Nilotinib	PDGFR	Novartis Bcr-Abl	2004	Chronic myelocytic leukemia	Fatigue, diarrhea, anorexia, skin discoloration, rash, hand-foot syndrome, edema, muscle cramps, joint pain, headache, abdominal discomfort, anemia, cough and itching, heart failure, pancreatitis, kidney failure
	Sorafenib	VEGFR, PDGER	Bayer Raf	2005	Renal cell carcinoma	Diarrhea, fatigue, hair loss, constipation, skin rash, high blood pressure
	Sunitinib	PDGFR, VEGFR	Pfizer	2006	Renal cell carcinoma	Hand and foot skin reactions, rash, diarrhea, fatigue, increased blood pressure, mucositis, fever, yellow skin, edema
	Dasatinib	SRC, PDGFR	Bristol-Myers Squibb Bcr-Abl	2006	Chronic myelocytic leukemia	Diarrhea, headache, nausea, rash, dyspnea, bleeding, fatigue, musculoskeletal pain, infection, vomiting, cough, abdominal pain, fever
	Lapatinib	EGFR	GlaxoSmithKline	2007	Breast cancer	Nausea, diarrhea, stomatitis and indigestion, dry skin, rash, breathing difficulties and insomnia
	Pazopanib	VEGFR, PDGFR, FGFR	GlaxoSmithKline	2009	Renal cell carcinoma, soft tissue sarcoma,Nonsmall-cell lung cancer	Diarrhea, high blood pressure, hair color changes, nausea, anorexia, vomiting
	Crizotinib	ALK	Pfizer	2011	Nonsmall-cell lung cancer	Abnormal vision, nausea, diarrhea, vomiting, constipation, edema, fatigue
	Vandetanib	VEGFR, EGFR	AstraZeneca	2011	Thyroid cancer	Diarrhea, skin rash, acne, nausea, high blood pressure, headache, fatigue, loss of appetite, abdominal pain
	Axitinib	VEGFR	Pfizer	2012	Renal cell carcinoma	Diarrhea, high blood pressure, fatigue, loss of appetite, nausea, dysphonia, weight loss, vomiting, fatigue, constipation
	Afatinib	EGFR	Boehringer	2013	Nonsmall-cell lung cancer	Diarrhea, skin rash, stomatitis, paronychia, loss of appetite, nose bleeding, dry skin
	Erlotinib	EGFR	Roche	2013	Nonsmall-cell lung cancer	Skin rash, diarrhea, loss of appetite, fatigue, dyspnea, cough, nausea, infection, vomiting, stomatitis, itching, dry skin, conjunctivitis, keratoconjunctivitis, abdominal pain
	Ceritinib	ALK	Novartis	2014	Nonsmall-cell lung cancer	Diarrhea, nausea, vomiting, abdominal pain, fatigue, loss of appetite, constipation
	Osimertinib	EGFR	AstraZeneca	2015	Nonsmall-cell lung cancer	Skin rash, mouth ulcers, paronychia
	Regorafenib	VEGFR, EGFR	Bayer	2017	Colorectal cancer, Hepatocellular carcinoma,Gastrointestinal Stromal Tumors	Fatigue, loss of appetite, diarrhea, oral mucositis, weight loss, high blood pressure, dysphonia.
	Lorbrena	ALK	Pfizer	2018	Nonsmall-cell lung cancer	Edema, cognitive effects, dyspnea, fatigue, weight gain, joint pain, diarrhea
	Dacomitinib	EGFR/HER2/HER4	Pfizer	2018	Nonsmall-cell lung cancer	Diarrhea, skin rash, paronychia, stomatitis
	Cabozantinib	MET/VEGFR1/VEGFR2/VEGFR3/ROS1/RET/AXL/NTRK/KIT	Exelixis		Medullarythyroidcance (2012) Renal cell carcinoma (2016) Hepatocellular carcinoma (2018)	Diarrhea, stomatitis, weight loss, loss of appetite, nausea, fatigue, oral pain, changes in hair color, dysgeusia, high blood pressure, abdominal pain, constipation

## 3 Tumor-Specific Endothelial Cells

At present, tumor angiogenesis research and antiangiogenic drug development use cultured ECs, such as human umbilical vein ECs (HUVECs). A number of studies have clarified the molecular differences between TECs and NECs through global analysis and compare TECs with NECs to try to find specific molecules for TECs. For example, Alam et al. conducted a DNA chip analysis and found that suprabasin might be a new marker for TECs. Compared with NECs, suprabasin, the upstream factor of the AKT pathway, was highly expressed in TECs and positively correlated with the migration and tube formation ability of TECs (50). Microarray and immunohistochemical analyses revealed that biglycan was a specific marker and an autocrine angiogenic factor of TECs (51). Goveia et al. performed single-cell RNA (scRNA) sequencing on 56,771 ECs from human/mouse tumor in lungs and cultured human lung TECs, and detected 17 known and 16 previously unrecognized phenotypes. And found that collagen modification was a candidate pathway for angiogenesis (52). Among the abovementioned studies, a few have focused on the function of TECs because human primary TECs culture have several limits, including small amounts from surgical specimens, difficult to separate, a short life span *in vitro*, easily lose their specificity and cannot be cultured in large quantities. In 2019, NakoMaishi and others established immortalized human TECs (h-imTECs) and their normal counterparts (h-imNECs) by transfecting with a lentivirus that produces simian virus 40 large T antigens and human telomerase reverse transcriptase to overcome replication barriers. These ECs exhibited an extended life span and retained their characteristic endothelial morphology, endothelial marker expression, and tube formation ability. Hence, these h-imTECs could be a valuable tool for drug screening to develop novel therapeutic agents specific to TECs or functional biological assays in tumor angiogenesis research (53).

### 3.1 Heterogeneity of Tumor-Specific Endothelial Cells

As a component of blood vessels, TECs are also different from NECs in many aspects (54). The whole tumor can be heterogeneous and biopsy may not be representative of the whole tumor. Angiogenesis contributes to the development of pathological conditions, such as tumor progression and metastasis, diabetic retinopathy, psoriasis, atherosclerosis, and rheumatoid arthritis (55).

As a component of blood vessels, TECs are also different from NECs in many aspects. Compared with most normal ECs, TECs have a higher proliferation rate and do not form a regular monolayer; the tumor vascular basement membrane is discontinuous or nonexistent, and the tumor endothelium is variably covered by pericytes with abnormal morphology (56, 57). Due to the phenotypic difference between tumor blood vessels and normal blood vessels, studies have concluded that may also exist genotype changes. Kyoko Hida found that the nucleus of TECs was larger than that of NECs and showed increased aneuploidy, abnormal centrosomes, and abnormalities, such as chromosome deletion, markers of unknown origin, and double microchromosomes (58). Chromosomal aberrations are also found in human renal carcinoma ECs and human B-cell lymphoma microvascular ECs (59, 60). These findings can be used as evidence of genetic instability of TECs. High-throughput expression profiles reveal changes in gene and protein expression profiles in the tumor endothelium. Li C et al. analyzed the expression differences between cervical cancer-derived ECs and NECs by scRNA-seq, and found several marker genes such as TAGLN2, KLF5, STAT1 and STAT2 (7). ScRNA sequencing revealed that 2590 genes were differentially expressed between TECs isolated from human hepatocellular carcinoma and NECs isolated from normal liver tissues (61). Proteomics analysis identified 127 highly expressed proteins in ECs isolated from human renal cancer, colon cancer, and lung cancer compared with NECs, among which CD146, CD31, and VWF might be tumor endothelial cell markers (62). In addition to expressing common vascular endothelial markers,TECs also show upregulated expression of VEGFR-2, VEGFR-3, e-selectin, ICAM-1, CD44, integrin and MUC-18 (63) and the nontraditional angiogenic factors biglycan, lysyl oxidase and pentraxin 3, which together promote tumor angiogenesis (64). These data indicate that the huge differences between TECs and NECs can be exploited to specifically target TECs for tumor treatment.

In addition to differing from NECs, different TECs also show heterogeneity. TECs have long been regarded as normal somatic cells without tumor characteristics, such as susceptibility to mutation and drug resistance. However, in glioblastoma, some ECs were found to originate from the glioblastoma stem cell population (65). In lymphoma and neuroblastoma, ECs derived from tumor cells were also confirmed (60, 66). In recent years, bone marrow-derived endothelial progenitor cells have been found to be involved in the formation of tumor pathological blood vessels, although the mechanism of action is still unclear (67). These findings provide new targets and directions for antitumor therapies that specifically target TECs but also increase the difficulty of developing such therapies.

**Figure 2:**
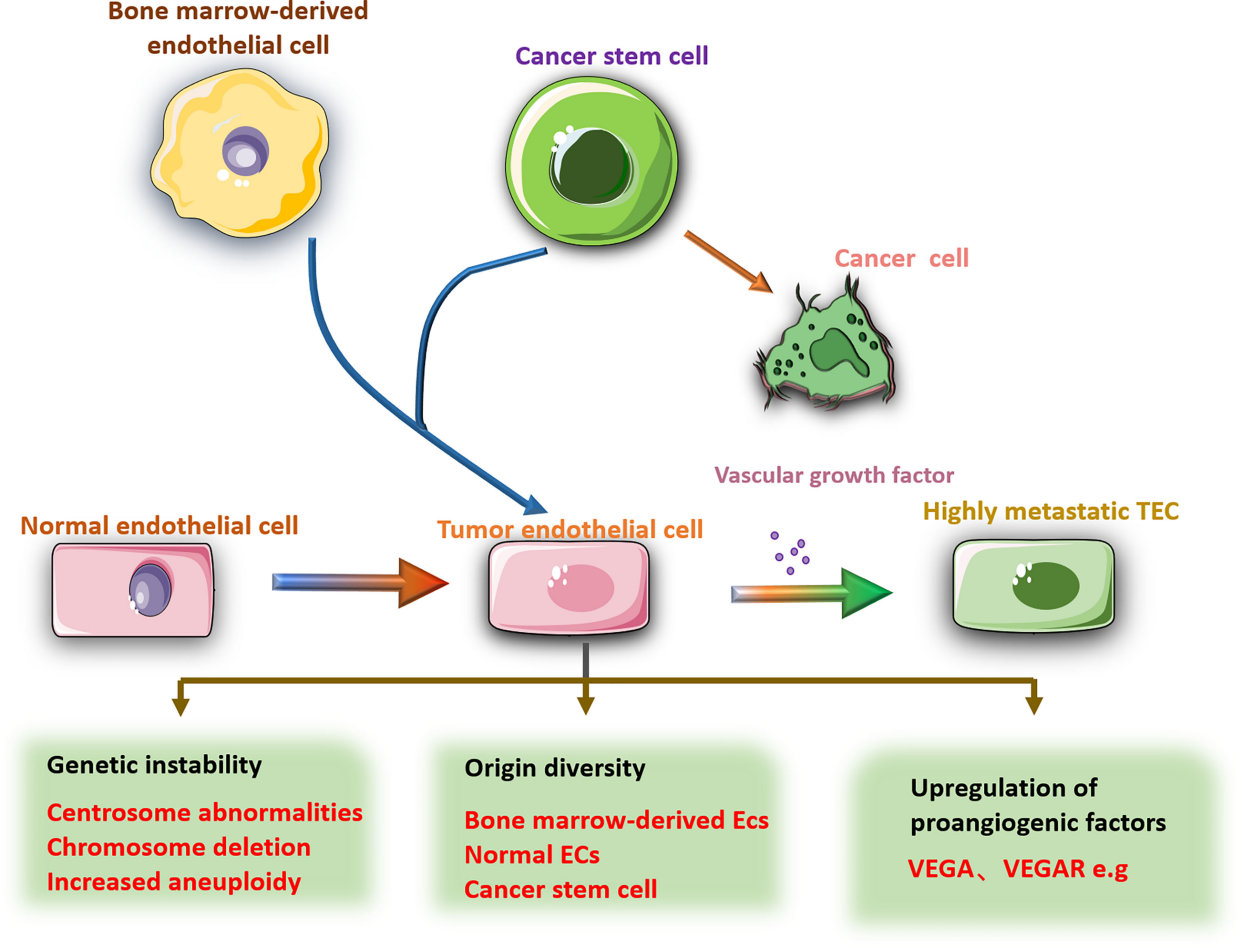
There are obvious differences between TECs and NECs. Compared with NECs, TECs have larger nuclei, increased aneuploidy, abnormal centrosomes, and chromosomal deletions. The source of TECs is diverse, including NECs mutations, bone marrow-derived ECs, cancer stem cells and other cell transformations. The gene changes lead to high expression of pro-angiogenic factors in TECs, such as VEGF, VEGFR, etc., which promote tumor angiogenesis. In addition, the phenotype of TECs is also affected by the tumor microenvironment.

In addition to the different origins of TECs, TEC phenotypes are affected by TME. Comparing the TECs stimulated by two different metastatic tumor supernatants, found that the treatment of highly metastatic tumor conditioned medium increased the resistance of TECs to 5-fluorouracil (5-FU) (68). After coculture with lung cancer cells, human umbilical vein ECs showed enhanced cell motility and microvascular formation and a decrease in the percentage of apoptosis (69). Conditional culture can also cause epigenetic changes in gene expression in cultured ECs (70). This evidence reveals the influence of the tumor microenvironment on the characteristics of TECs and leads to heterogeneity among TECs. Therefore, studying the heterogeneity and diversity of TECs in the development of tumors will contribute to the development of antitumor angiogenesis drugs. Antitumor research about specifically targeting TECs has become a trend (**Figure 2**).

### 3.2 Function of Tumor-Specific Endothelial Cells in Cancer Progression

#### 3.2.1 Tumorigenesis

The cellular origin of cancer and the nature of cells responsible for the maintenance and progression of tumors are still unsolved challenges for cancer therapy (71). Predictably, cancer originating from a single cell expands with the development of the cancer (72). Cancer stem cells (CSCs) have stem cell-like properties and can renew themselves. A number of studies have found that CSCs may cause cancer (73), thus leading to tumor recurrence (74). In melanoma,ECs can interact with CD133+ cancer stem cells to promote the occurrence and development of tumors (75). Targeting Cxcl12+ECs can inhibit the formation of the niche of gastric stem cells around blood vessels and inhibit the occurrence of diffuse gastric cancer (76). The specific regulatory mechanism of TECs on tumor stem cells is not very clear. *In vitro* experiments found that HUVECs co-cultured with human liver cancer cells (MHCC97H) enhanced the spheroidizing ability of MHCC97H cells and the expression of CD133 (77). Jia et al. found that TECs could release soluble factors through paracrine action and increasedthe CSCs ratio, clonal sphere formation, tumorigenicity and chemoresistance (78). These effects are caused by the activation of Notch signaling in TECs and changes in the CSCs phenotype (78, 79). Knocking out the specific Notch ligand Dll4 inECs inhibits Epithelial-Mesenchymal Transition(EMT) and results in a reduction in the number of CSCs and decreased tumor metastasis (80). The above evidence shows that TEC may regulate cancer stem cells through the Notch signaling pathway. In addition, studies have found that TECs may also regulate the phenotype and chemotherapy resistance of CSCs through the AKT, Src, and FAK signaling pathways (81, 82).

**Figure 3:**
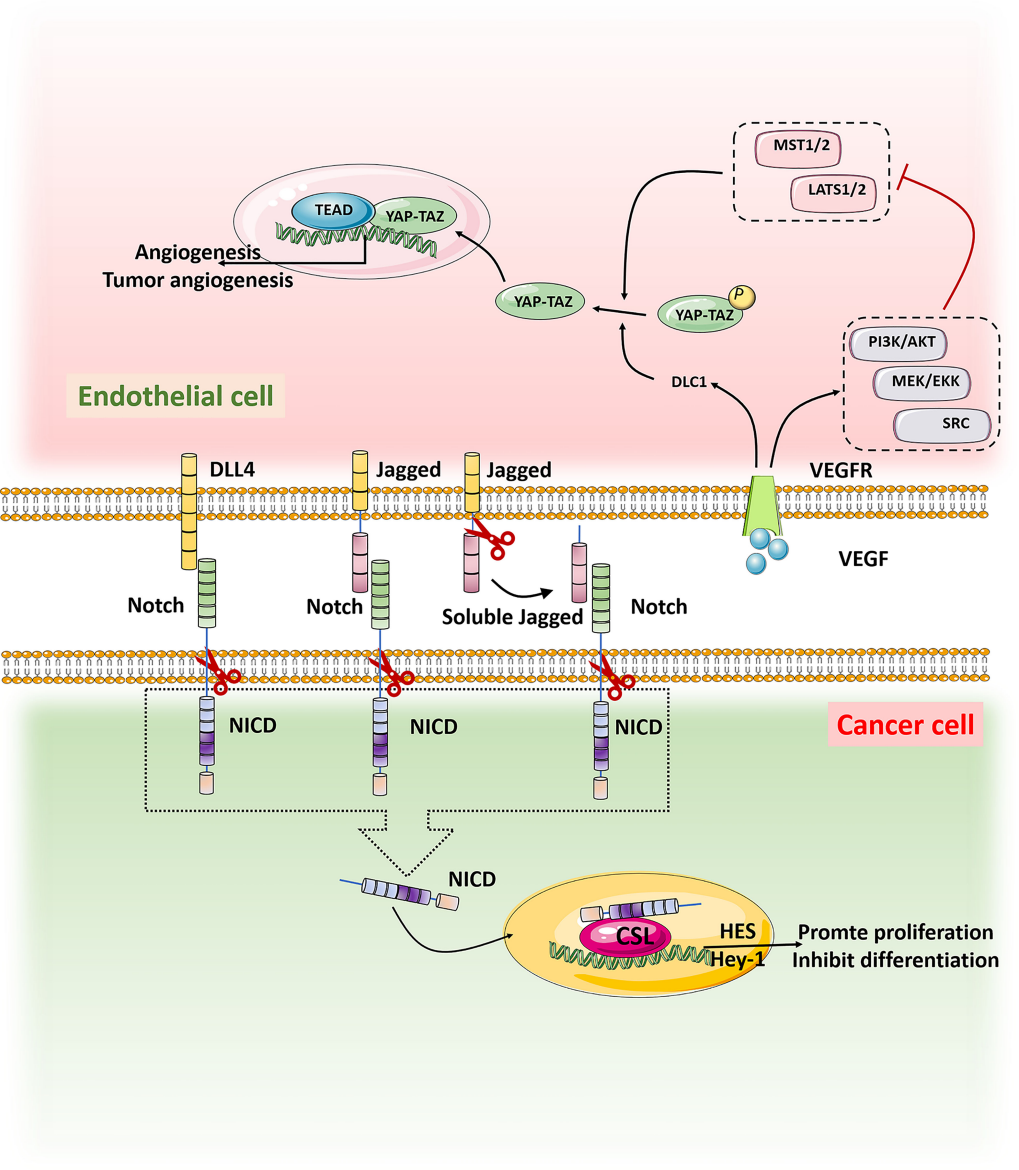
When Notch on tumor cells binds to ligands DLL4 and Jagged on TECs, it is activated and cut by Y secretion to release the Notch intracellular domain NICD, which is translocated into the nucleus by activating transcriptional targets HES and Hey1 to inhibit differentiation and maintain stemness. VEGF in the tumor microenvironment binds to the receptor VEGFR on TECs, promoting the expression of tumor suppressor gene DLC1 and the activation of signaling pathways such as PI3K/AKT, MEK/EKK and SRC, thus inhibiting the activation of MST1/2-LATS1/2 kinase cassette. YAP/TAZ was dephosphorylated and translocated into the nucleus. In the nucleus, YAP/TAZ interacts with the transcription factor TEAD to promote tumor genesis and angiogenesis.

The transcriptional regulator YAP/TAZ has strong activity in malignant tumors and has been found to promote the occurrence of many tumors, including gastric cancer, colorectal cancer, liver cancer, and neuroblastoma. The YAP/TAZ signaling could influence the function of ECs to promote the tumor progression (83). Up-regulation of the tumor suppressor gene DLC1 in ECs activated YAP signal and ECs lost the contact inhibition function,which leaded to the occurrence and proliferation of angiosarcoma (84, 85). YAP also controls the activation of ECs and regulates tumorigenesis and angiogenesis (86). Thus, interference with the YAP/TAZ signaling pathway is expected to suppress tumorigenesis and tumor angiogenesis (**Figure 3**).

#### 3.2.2 Tumor Transendothelial Migration

**Figure 4:**
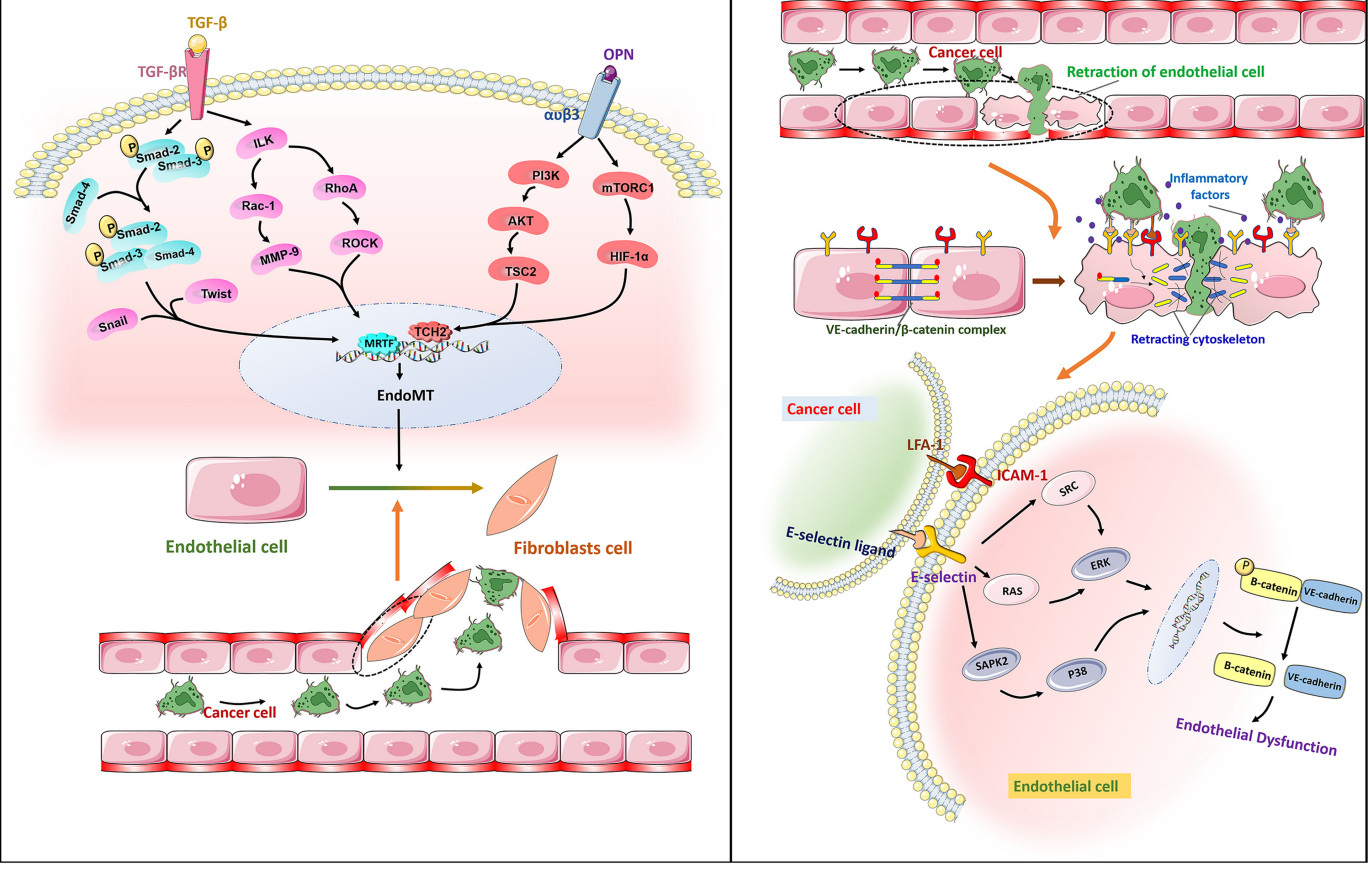
Right: Free TGF-β binds to receptors on the surface of ECs membranes, phosphorylates Smad2 and Smad3, then binds to Smad4, enters the nucleus, and binds to Snail, Twist and other transcription factors to initiate EndoMT by promoting transcription of mesenchymal markers and reducing transcription of endothelial markers. It leads to endothelial dysfunction and promotes the transendothelial migration of cancer cells. Osteopontin (OPN) interacts with a variety of integrins. The combination of OPN and α V β3 activates PI3K/AKT and mTORC1 pathways and promotes the metastasis of cancer cells.Left: The adhesion molecule ICAM-1 binds to e-selectin through ligand receptor, which enables tumor cells to bind to endothelial cells. The dissociation of the VE-cadherin/β -catenin complex is associated with endothelial barrier dysfunction. E-selectin regulates the transendothelial migration of cancer cells by activating the ERK and P38 signaling pathways and mediating the dissociation of the VE-cadherin/β -catenin complex.

Metastasis is the main cause of cancer patient death, which has not yet been resolved by current tumor treatments. Tumor cell migration across the endothelium is an important step in the process of tumor invasion and metastasis. Tumor cells cross the basement membrane and enter peripheral blood circulation to reach distant organs, then adhere to vascular ECs through cell adhesion molecules, and migrate through vascular endodermis to colonize these organs. Endothelial-to-mesenchymal transition (EndoMT) is considered a necessary process for tumor migration across the endothelium. EndoMT is a complex cell differentiation process in which ECs break away from the cell population and migrate, which reduces the characteristics of ECs to varying degrees, and these cells then acquire mesenchymal characteristics. The main hallmark of EndoMT is that TGF-β inducesECs to transform into CAF-like cells, thus leading to the loss of endothelial adhesion molecules and endothelial cytoskeleton reorganization through the Rho and Rac-1 signaling pathway (87) (**Figure 4**). EndoMT helps to destroy the endothelial barrier, which leads to tumor extravasation and increase metastasis. Studies have confirmed that EndoMT increases the transendothelial migration of melanoma (88). Similarly, EndoMT, which is mediated by osteopontin (OPN) through the PI3K/Akt/TSC2 and mTORC1 signaling pathways, promotes the growth and metastasis of colorectal cancer (89). EndoMT caused by endoglin deficiency causeincreased liver and lung metastasis in pancreatic cancer model mice (90). Therefore, it is feasible to use EndoMT as a target to inhibit tumor metastasis. Moreover, some studies have pointed out that EndoMT may be the cause of drug resistance in antitumor therapy (91). Therefore, the role of EndoMT needs to be explored and fully understood.

Cell adhesion molecules are essential for tumor migration across the endothelium. Laferriere et al. found that E-selectin expressed by TECs ensured that colon cancer cells adhered to ECs and activated the SAPK2/P38 signaling pathway to promote tumor transendothelial migration (92). In addition, the activation of ERK by E-selectin regulates the opening of the endothelial space by initiating the activation of Src kinase activity and the dissociation of the VE-cadherin/β-catenin complex (93). In melanoma, ICAM-1 expressed in TECs interacts with its receptor integrin LFA-1 to promote tumor cell migration across the endothelium *in vitro (* 94). The VE-cadherin binding domain of fibrinogen induces the permeability of the endothelial barrier and enhances the transendothelial migration of malignant breast epithelial cells (95). Therefore, blocking the production of cell adhesion molecules or inhibiting their function has great potential to inhibit tumor metastasis. Studies have confirmed that anti-cell adhesion molecule antibodies can inhibit tumor growth and metastasis. For example, after treatment with an anti-L1CAM antibody, the cancer growth (up to 75%) of SKOV3ip ovarian cancer cells was significantly reduced (96). Unfortunately, such treatment cannot effectively eliminate highly malignant tumors. In view of this situation, additional in-depth research is required to find more effective treatment strategies that target cell adhesion molecules (**Figure 4**).

#### 3.2.3 Tumor Resistance

Drug resistance is an obstacle that impairs the success of cancer therapies. In some cases, relapse occurs in initially responsive patients after repeated cycles of chemotherapy due to the acquisition of tumor resistance (97). In the early stage, TECs were considered to be homogenous, these genetic stable cell populations did not cause drug resistance. With the progression of tumor, as we previously mentioned, TECs occurred considerable heterogeneity and genetic instability which might lead to drug resistance (98). A number of experiments have proven that ECs show resistance to some drugs. For example, kidney cancer ECs are resistant to vincristine (63) while liver cancer ECs are resistant to adriamycin (99) and 5-fluorouracil (100). However, it is not clear whether this resistance is related to the genomic characteristics of TECs. The resistance of ECs to antiangiogenic treatment appears to be related to the increased expression of multidrug resistance proteins, such as P-glycoprotein (Pgp, ABCB1) and breast cancer resistance protein (BCRP,ABCG2), which serve as cellular efflux pumps (101, 102). In addition, Ca2+ transporters are also changed in stromal cancer cells, including ECs and endothelial colony forming cells (103). The remodeling of the endothelial Ca2+ toolkit may enhance the resistance of anticancer treatments by supporting tumor angiogenesis and reducing the sensitivity to proapoptotic stimuli (104). VEGF is an important target for antitumor angiogenesis. Studies have found that the failure of anti-VEGF drugs in anticancer treatment may be due to the recruitment of endothelial progenitor cells. VEGF inhibitors can induce the expression of placental growth factor, IL-6 and stem cell factors in nontumor tissues, and these cytokines can recruit bone marrow-derived ECs and myeloid progenitor cells to promote the formation of a premetastatic environment. Some of these recruited cells express VEGFR-1 and are resistant to VEGF inhibitors that target VEGFR-2 (105).

### 3.3 Interaction of Tumor-Specific Endothelial Cells and the Tumor Microenvironment

**Figure 5:**
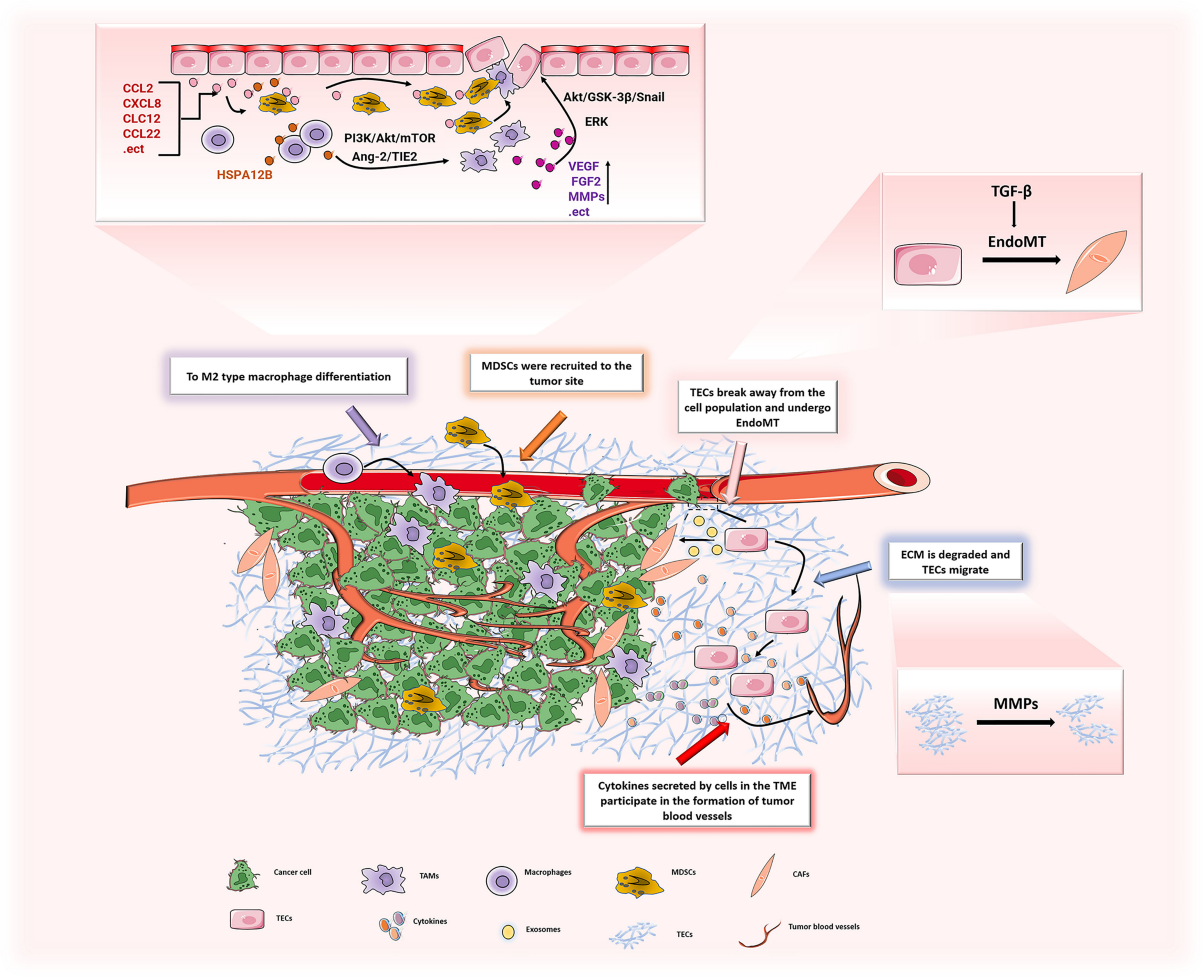
In TME, the various components interact to promote the development of ECs in the direction of promoting angiogenesis. ECs can induce M2 polarization of macrophages through PI3K/Akt/mTOR, and may recruit TAMs to tumor sites through the Ang-2/TIE2 signaling pathway to promote tumor progression. TAMs secrete a large number of pro-angiogenic factors (VEGF, FGF2, MMPs, etc.) to promote the proliferation of ECs, leading to tumor angiogenesis. The chemokines (CCL2, CXCL8, etc.) secreted by ECs recruit MDSCs to the tumor and play a tumor-promoting effect. ECs generate CAFs through EndoMT, and the generated CAFs secrete pro-angiogenic factors to stimulate ECs and promote tumor angiogenesis. After ECM is degraded by MMPs, it increases the migration of ECs and promotes tumor angiogenesis.

The interaction between a tumor and mesenchymal cells may be the reason for the abnormal structure of mesenchymal cells. The TME consists of stromal cells (including fibroblasts, macrophages, regulatory T cells, myeloid suppressor cells, ECs, pericytes and platelets) and extracellular matrix components (including inflammatory cytokines, chemokines and Matrix metalloproteinases), which enhance invasion and metastasis of cancer cells through mutual signal transduction. The TME induces gene expression in ECs to develop in a direction that is conducive to angiogenesis (**Figure 5**).

#### 3.3.1 Tumor-Associated Macrophages

TAMs are the key cells that control tumor angiogenesis and the main source of angiogenic factors. TAMs can secrete angiogenesis factors, such as VEGF (106–108), basic fibroblast growth factor (Fgf2) (109), insulin-like growth factor-1 (Igf1) (110), chemokine ligand 2 (Ccl2) (111, 112) and placental growth factor (Pgf) (113)ect. These factors can stimulate ECs and promote proliferation rapidly of cells, which leads to tumor angiogenesis (14). Recent studies have found that TAMs highly express certain cytokines in gastric cancer, such as VEGF-A, VEGF-C, matrix metalloproteinase 1 (MMP-1) and amphiregulin (114), and induces capillary morphogenesis in human gastric cancer lymphatic ECs (115). Another study showed that CCL18 released by TAMs cooperated with VEGF to promote the migration of ECs, induce EndoMT, and activate ERK and Akt/GSK-3β/Snail signals in HUVECs, thereby promoting breast cancer angiogenesis (116). The latest breakthrough study found that an M1-like macrophage subtype might keep vascular cells quiescent, and at the same time, Matrix-remodeling macrophages might assist invasive cancer cells to co-opt vessels (117). Therefore, TAMs regulate the phenotype and function of ECs in the process of tumor angiogenesis and vascular remodeling. There is no doubt that TECs can also act on TAMs. First, TECs can recruit TAMs to tumor sites, which are mediated by several signaling pathways including the Ang-2/TIE2 signaling (118, 119). Second, changes in the permeability ofECs will also increase the infiltration of TAMs, thereby promoting the development of tumors (120). In addition, studies have found that ECs could selectively activate the differentiation of tumor-promoting M2 macrophages and promoted tumor angiogenesis (121). The latest research confirms that TECs may induce M2 polarization of macrophages by secreting HSPA12B to activate the PI3K/Akt/mTOR signal pathway (122). The above evidence shows that ECs play a key role in inducing the differentiation of macrophages and promoting further polarization to a pro-angiogenic phenotype. Therefore, studying the interaction between TECs and TAMs may provide a novel therapeutic approach on specifically targeting TECs.

#### 3.3.2 Tumor-Associated Fibroblasts

In the TME, CAFs are a major matrix component that helps build the extracellular matrix and provides necessary growth factors for tumor cell growth and development. There are various sources of CAFs, such as activated tissue cells, transdifferentiated pericytes and adipocytes. However, CAFs can also be generated from transdifferentiated ECs through EndoMT (123). Studies have speculated that approximately 40% of CAFs are formed from ECs (124). As mentioned above, this process is mainly mediated by TGF-β. TGF-β secreted by tumor cells stimulates the phosphorylation of TGF-β receptors on the surface of ECs and activates Smad, which in turn activates the downstream signal transduction cascade, then leads to the occurrence of EndoMT and to generate CAFs (125, 126). A study found that exosomes secreted by tumors may also mediate the differentiation of ECs into CAFs and promote tumor invasion (123). Another study confirmed that melanoma-derived exosomes increased the number of CAFs differentiated from HUVECs by promoting EndoMT, which showed obvious morphological, molecular changes and motility (127). It is worth mentioning that the generated CAFs secrete a large number of cytokines, which can react with TECs and promote tumor angiogenesis. For example, CAFs release a large amount of angiogenic factors, such as VEGF-A (128) and FGF2 (129), in the TME to activate ECs and promote tumor angiogenesis. Interestingly, CAFs can also recruit endothelial progenitor cells by secreting CXCL12 to accelerate tumor growth and increase angiogenesis (130). In addition, early studies have found that platelet-derived growth factor C (PDGF-C) produced by CAFs can act on ECs and enhance the resistance to anti-angiogenesis and anti-VEGF treatments (131). Later studies showed that CAFs enhanced the motility and permeability of ECs by upregulating the LPP gene and promoted chemotherapy resistance in ovarian cancer (132). Overall, studies on the relationship between TECs and CAFs will provide a deeper understanding of tumor angiogenesis and chemotherapy drug resistance. Inhibiting the generation of CAFs or killing existing CAFs might represent effective therapeutic targets for antitumor angiogenesis.

#### 3.3.3 Myeloid-Derived Suppressor Cells

MDSCs are immature myeloid cells that are normally produced and secreted by the bone marrow in the state of local inflammation. They have strong immunosuppressive activity, which can inhibit excessive inflammation and protect the host from autoimmune diseases (133). In the tumor state, MDSCs are abnormally produced and recruited to the TME to help establish an immunosuppressive TME and promote tumor angiogenesis and metastasis to support tumor progression. There are two main types of MDSCs, mononuclear MDSCs (M-MDSCs) and polymorphonuclear MDSCs (PMN-MDSCs) (134). In tumors, M-MDSC can quickly differentiate into TAMs (135, 136), and the relationship between TAMs and ECs and the promotion of tumors have been discussed above. However, evidence has shown that PMN-MDSCs are mainly recruited to tumor tissues by chemokines to play a pro-tumor effect. Sushil et al. found that CXCL2 and CCL22 promoted MDSC recruitment to primary tumors and metastatic sites in triple-negative breast cancer (137). However, inhibiting the expression of CXCL1 and CXCL2 reduces the recruitment of MDSCs in ovarian cancer (138). ECs are the main sources of chemokines. Studies have shown that in a variety of tumors, TECs can secrete a large number of chemokines, such as CCL2, CXCL8 and CXCL12 (139, 140). Chemokine receptors are distributed on the cell membrane of MDSCs, and chemokine recruit MDSCs to the tumor site by binding these chemokine receptors and promote tumor progression (141). After knocking down the chemokine receptor (CXCR2) in myeloid cells, the recruitment of MDSCs is reduced and vascular remodeling is inhibited (142). The above studies show that TECs play a key role in MDSCs recruitment and tumor progression. Similarly, MDSCs can also regulate TECs. A study found that MDSCs may interact with lysosomal acid lipase to cause dysfunction of ECs (16). Moreover, *in vitro* experiments have shown that the culture supernatant of PMN-MDSCs can significantly promote the tube formation of HUVECs (143). This finding indicates that PMN-MDSCs may secrete some pro-angiogenic factors. Later studies confirmed that MDSCs might promote endothelial cell angiogenesis through the production of VEGF-A, Ang2 and HIF-1α and unregulated angiogenesis by activating the STAT3 signaling pathway (144). In summary, TECs play key roles in the pro-tumor effect of MDSCs and exploring the mechanisms involved in MDSCs-induced tumor angiogenesis will provide new insights for anti-angiogenesis therapy.

#### 3.3.4 Extracellular Matrix

Along with stromal cells, the extracellular matrix is another important part of the TME. The extracellular matrix (ECM) is a noncellular three-dimensional polymer network composed of collagen, elastin, fibronectin, laminin and other proteins. It can regulate a variety of cellular functions and is essential for maintaining normal homeostasis. The production and maintenance of the ECM is an important aspect of endothelial cell function. The ECM provides mechanical support to ECs and mediates signaling (145) *via* secreted molecules (146) and mechanical strain (147)between cells. In the absence of angiogenesis stimulation, ECM helps ECs to maintain in a quiescent state. In the tumor growth stage, the ECM is degraded in the TME and the basement membrane is destroyed, which cause ECs migrate from existing blood vessels to newly formed blood vessels. In this progress, MMPs play a major role. MMPs are the main enzyme in degradatingextracellular matrix proteins, and can participate in the occurrence and development of tumors in different ways. On the one hand, MMPs can regulate the expression of VEGF to promote formation of neovascularization and increase blood vessel permeability (148). Moreover, MMPs can also degrade collagen to promote the migration of ECs, which adhere to the temporary ECM through specific integrins to form a tubular structure and obtain a continuous lumen (149). On the other hand, it can degrade a variety of extracellular matrix components to promote tumor progression. For example, MMP-2 promotes the transendothelial migration of breast cancer cells by degrading the laminin component of the ECM (150). MMP9 can bind to CD44 to degrade fibronectin, which leads to the active form of TGF-βreleasing (151), then TGF-β enhances the conversion of ECs to endothelial mesenchyme and increases the quantity of CAFs to promote tumor progression (152). In short, the ECM is the main regulator of angiogenesis and vascular stability. However, it is not a stable component and will undergo tremendous changes under a variety of pathological conditions including tumors. Therefore, promoting the normalization of ECM composition can be used as a new therapy for antitumor angiogenesis.

## 4 Conclusion

This review summarizes the tissue differences between tumor blood vessels and normal blood vessels. Tumor blood vessels are highly irregular in shape with increased vascular permeability and leakage, which contributes to the tumor metastasis. Contrary to previous ideas, recent studies suggest that TECs differ from NECs in many aspects. Different studies have revealed TECs originate from various cell types, for example bone marrow-derived endothelial progenitor cells and tumor cells. The development of TECs and angiogenesis are affected by the intricate TME including infiltrated various cells and inflammatory cytokines, growth factors and so on. All the above described factors can contribute to the different gene expression profile between TECs and NECs, which affects the formation and function of tumor blood vessels. Moreover, identification of relevant factors influencing the structural and functional differences between TECs and NECs are also imminent to provide clues for target therapies of angiogenesis. Taken together, this review summarizes the most recent research developments in the field of the molecular and cellular features of angiogenesis of cancer biology. Importantly, this review systematically introduces the current knowledge on TECs, and provides new insights into the potential of targeted therapies.

## Author Contributions

JL drafted the manuscript, SW and GZ drew the figures, BH contributed equally to plot the table, BZ and QB revised the review. All authors contributed to the article and approved the submitted version.

## Funding

This study was supported by grants from the National Natural Science Foundation of China (No. 82173371, 81802446), project tsqn201909192 supported by Tai Shan Young Scholar Foundation of Shandong Province, project ZR2019BH050, ZR2020YQ59, ZR202103020202 supported by Shandong Provincial Natural Science Foundation; PhD Research Foundation of the Affiliated Hospital of Jining Medical University (No. 2018-BS-001, 2018-BS-013), project 202003031182, 202003031183 supported by the Project of Medicine Health and Technology Development Plan of Shandong Province, project CX2020081, CX2020042, CX2020035 supported by University Student Innovation Training Program of Jining Medical University, the Miaopu Research of the Affiliated Hospital of Jining Medical University (No. MP-ZD-2020-005).

## Conflict of Interest

The authors declare that the research was conducted in the absence of any commercial or financial relationships that could be construed as a potential conflict of interest.

## Publisher’s Note

All claims expressed in this article are solely those of the authors and do not necessarily represent those of their affiliated organizations, or those of the publisher, the editors and the reviewers. Any product that may be evaluated in this article, or claim that may be made by its manufacturer, is not guaranteed or endorsed by the publisher.

## References

[1] Brown LF Guidi AJ Schnitt SJ Van De Water L Iruela-Arispe ML Yeo TK. Vascular Stroma Formation in Carcinoma *in Situ*, Invasive Carcinoma, and Metastatic Carcinoma of the Breast. Clin Cancer Res (1999) 5:1041–56. 10353737

[2] Tilki D Kilic N Sevinc S Zywietz F Stief CG Ergun S. Zone-Specific Remodeling of Tumor Blood Vessels Affects Tumor Growth. Cancer (2007) 110:2347–62. doi: 10.1002/cncr.23024 17849463

[3] Viallard C Audiger C Popovic N Akla N Lanthier K Legault-Navarrete I. BMP9 Signaling Promotes the Normalization of Tumor Blood Vessels. Oncogene (2020) 39:2996–3014. doi: 10.1038/s41388-020-1200-0 32042114

[4] Lin PP. Aneuploid Circulating Tumor-Derived Endothelial Cell (CTEC): A Novel Versatile Player in Tumor Neovascularization and Cancer Metastasis. Cells (2020) 9:1539. doi: 10.3390/cells9061539 PMC7349247 32599893

[5] Dhanabal M Jeffers M Larochelle WJ. Anti-Angiogenic Therapy as a Cancer Treatment Paradigm. Curr Med Chem Anticancer Agents (2005) 5:115–30. doi: 10.2174/1568011053174882 15777219

[6] Nagl L Horvath L Pircher A Wolf D. Tumor Endothelial Cells (Tecs) as Potential Immune Directors of the Tumor Microenvironment - New Findings and Future Perspectives. Front Cell Dev Biol (2020) 8:766. doi: 10.3389/fcell.2020.00766 32974337 PMC7466447

[7] Li C Guo L Li S Hua K. Single-Cell Transcriptomics Reveals the Landscape of Intra-Tumoral Heterogeneity and Transcriptional Activities of Ecs in CC. Mol Ther Nucleic Acids (2021) 24:682–94. doi: 10.1016/j.omtn.2021.03.017 PMC8099483 33996252

[8] Pfisterer J Shannon CM Baumann K Rau J Harter P Joly F. Bevacizumab and Platinum-Based Combinations for Recurrent Ovarian Cancer: A Randomised, Open-Label, Phase 3 Trial. Lancet Oncol (2020) 21:699–709. doi: 10.1016/S1470-2045(20)30142-X 32305099

[9] Zhao B Zhao H Zhao J. Risk of Fatal Adverse Events in Cancer Patients Treated With Sunitinib. Crit Rev Oncol Hematol (2019) 137:115–22. doi: 10.1016/j.critrevonc.2019.03.007 31014507

[10] Ni Y Zhou X Yang J Shi H Li H Zhao X. the Role of Tumor-Stroma Interactions in Drug Resistance Within Tumor Microenvironment. Front Cell Dev Biol (2021) 9:637675. doi: 10.3389/fcell.2021.637675 34095111 PMC8173135

[11] Yamaguchi R Perkins G. Animal Models for Studying Tumor Microenvironment (TME) and Resistance to Lymphocytic Infiltration. Cancer Biol Ther (2018) 19:745–54. doi: 10.1080/15384047.2018.1470722 PMC6154837 29723108

[12] Luis G Godfroid A Nishiumi S Cimino J Blacher S Maquoi E. Tumor Resistance to Ferroptosis Driven by Stearoyl-Coa Desaturase-1 (SCD1) in Cancer Cells and Fatty Acid Biding Protein-4 (FABP4) in Tumor Microenvironment Promote Tumor Recurrence. Redox Biol (2021) 43:102006. doi: 10.1016/j.redox.2021.102006 34030117 PMC8163990

[13] Sun M Qiu S Xiao Q Wang T Tian X Chen C. Synergistic Effects of Multiple Myeloma Cells and Tumor-Associated Macrophages on Vascular Endothelial *Cells in Vitro* . Med Oncol (2020) 37:99. doi: 10.1007/s12032-020-01426-1 33040185

[14] Jetten N Verbruggen S Gijbels MJ Post MJ De Winther MP Donners MM. Anti-Inflammatory M2, But Not Pro-Inflammatory M1 Macrophages Promote Angiogenesis *in Vivo* . Angiogenesis (2014) 17:109–18. doi: 10.1007/s10456-013-9381-6 24013945

[15] Pausch TM Aue E Wirsik NM Freire Valls A Shen Y Radhakrishnan P. Metastasis-Associated Fibroblasts Promote Angiogenesis in Metastasized Pancreatic Cancer *via* the CXCL8 and the CCL2 Axes. Sci Rep (2020) 10:5420. doi: 10.1038/s41598-020-62416-x 32214219 PMC7096431

[16] Zhao T Ding X Du H Yan C. Myeloid-Derived Suppressor Cells Are Involved in Lysosomal Acid Lipase Deficiency-Induced Endothelial Cell Dysfunctions. J Immunol (2014) 193:1942–53. doi: 10.4049/jimmunol.1301941 PMC4119579 25000979

[17] Bagulho A Vilas-Boas F Pena A Peneda C Santos FC Jeronimo A. the Extracellular Matrix Modulates H2O2 Degradation and Redox Signaling in Endothelial Cells. Redox Biol (2015) 6:454–60. doi: 10.1016/j.redox.2015.09.006 PMC4588420 26409032

[18] Folkman J. Tumor Angiogenesis: Therapeutic Implications. N Engl J Med (1971) 285:1182–6. doi: 10.1056/NEJM197111182852108 4938153

[19] Zhu CC Chen C Xu ZQ Zhao JK Ou BC Sun J. CCR6 Promotes Tumor Angiogenesis *via* the AKT/NF-Kappab/VEGF Pathway in Colorectal Cancer. Biochim Biophys Acta Mol Basis Dis (2018) 1864:387–97. doi: 10.1016/j.bbadis.2017.10.033 29097259

[20] Balamurugan K Koehler L Durig JN Hempel U Rademann J Hintze V. Structural Insights Into the Modulation of PDGF/PDGFR-Beta Complexation by Hyaluronan Derivatives. Biol Chem (2021) 402:1441–52. doi: 10.1515/hsz-2021-0173 34280958

[21] Ma J Tang W Gu R Hu F Zhang L Wu J. SHP-2-Induced Activation of C-Myc is Involved in PDGF-B-Regulated Cell Proliferation and Angiogenesis in Rmecs. Front Physiol (2020) 11:555006. doi: 10.3389/fphys.2020.555006 33329018 PMC7719712

[22] Mueller T Freystein J Lucas H Schmoll HJ. Efficacy of a Bispecific Antibody Co-Targeting VEGFA and Ang-2 in Combination With Chemotherapy in a Chemoresistant Colorectal Carcinoma Xenograft Model. Molecules (2019) 24:2865. doi: 10.3390/molecules24162865 PMC6719918 31394786

[23] Cheng W Cheng Z Weng L Xing D Zhang M. Asparagus Polysaccharide Inhibits the Hypoxia-Induced Migration, Invasion and Angiogenesis of Hepatocellular Carcinoma Cells Partly Through Regulating HIF1alpha/VEGF Expression *via* MAPK and PI3K Signaling Pathway. J Cancer (2021) 12:3920–9. doi: 10.7150/jca.51407 PMC8176233 34093799

[24] Wagner MJ Lyons YA Siedel JH Dood R Nagaraja AS Haemmerle M. Combined VEGFR and MAPK Pathway Inhibition in Angiosarcoma. Sci Rep (2021) 11:9362. doi: 10.1038/s41598-021-88703-9 33931674 PMC8087824

[25] Liao ZH Zhu HQ Chen YY Chen RL Fu LX Li L. the Epigallocatechin Gallate Derivative Y6 Inhibits Human Hepatocellular Carcinoma by Inhibiting Angiogenesis in MAPK/ERK1/2 and PI3K/AKT/HIF-1alpha/VEGF Dependent Pathways. J Ethnopharmacol (2020) 259:112852. doi: 10.1016/j.jep.2020.112852 32278759

[26] Ferrara N Hillan KJ Gerber HP Novotny W. Discovery and Development of Bevacizumab, an Anti-VEGF Antibody for Treating Cancer. Nat Rev Drug Discov (2004) 3:391–400. doi: 10.1038/nrd1381 15136787

[27] Zhao M Yu Z Li Z Tang J Lai X Liu L. Expression of Angiogenic Growth Factors VEGF, Bfgf and ANG1 in Colon Cancer After Bevacizumab Treatment in vitro: A Potential Self-Regulating Mechanism. Oncol Rep (2017) 37:601–7. doi: 10.3892/or.2016.5231 27840995

[28] Bamias A Gibbs E Khoon Lee C Davies L Dimopoulos M Zagouri F. Bevacizumab With or After Chemotherapy for Platinum-Resistant Recurrent Ovarian Cancer: Exploratory Analyses of the AURELIA Trial. Ann Oncol (2017) 28:1842–8. doi: 10.1093/annonc/mdx228 28481967

[29] Cheng Y Wu G Zhang S Liu Y Qu J Qu X. Complete Pathologic Response of Multiple Liver Metastases and Clinical Complete Response of Rectal Cancer in a Patient With Ataxia-Telangiectasia Mutated Gene Mutations After XELOXIRI Plus Bevacizumab: A Case Report. Onco Targets Ther (2021) 14:4201–9. doi: 10.2147/OTT.S320477 PMC8289441 34290508

[30] Ilic I Jankovic S Ilic M. Bevacizumab Combined With Chemotherapy Improves Survival for Patients With Metastatic Colorectal Cancer: Evidence From Meta Analysis. PloS One (2016) 11:e0161912. doi: 10.1371/journal.pone.0161912 27579775 PMC5006969

[31] Mahfouz N Tahtouh R Alaaeddine N El Hajj J Sarkis R Hachem R. Gastrointestinal Cancer Cells Treatment With Bevacizumab Activates a VEGF Autoregulatory Mechanism Involving Telomerase Catalytic Subunit Htert *via* PI3K-AKT, HIF-1Alpha and VEGF Receptors. PloS One (2017) 12:e0179202. doi: 10.1371/journal.pone.0179202 28594907 PMC5466359

[32] Brossa A Grange C Mancuso L Annaratone L Satolli MA Mazzone M. Sunitinib But Not VEGF Blockade Inhibits Cancer Stem Cell Endothelial Differentiation. Oncotarget (2015) 6:11295–309. doi: 10.18632/oncotarget.3123 PMC4484457 25948774

[33] Alamon C Davila B Garcia MF Sanchez C Kovacs M Trias E. Sunitinib-Containing Carborane Pharmacophore With the Ability to Inhibit Tyrosine Kinases Receptors FLT3, KIT and PDGFR-Beta, Exhibits Powerful *In Vivo* Anti-Glioblastoma Activity. Cancers (Basel) (2020) 12:3423. doi: 10.3390/cancers12113423 PMC7698965 33218150

[34] Tran TA Leong HS Pavia-Jimenez A Fedyshyn S Yang J Kucejova B. Fibroblast Growth Factor Receptor-Dependent and -Independent Paracrine Signaling by Sunitinib-Resistant Renal Cell Carcinoma. Mol Cell Biol (2016) 36:1836–55. doi: 10.1128/MCB.00189-16 PMC4911743 27141054

[35] Wang D Xiao F Feng Z Li M Kong L Huang L. Sunitinib Facilitates Metastatic Breast Cancer Spreading by Inducing Endothelial Cell Senescence. Breast Cancer Res (2020) 22:103. doi: 10.1186/s13058-020-01346-y 32993785 PMC7526390

[36] Carvalho B Lopes RG Linhares P Costa A Caeiro C Fernandes AC. Hypertension and Proteinuria as Clinical Biomarkers of Response to Bevacizumab in Glioblastoma Patients. J Neurooncol (2020) 147:109–16. doi: 10.1007/s11060-020-03404-z 31974803

[37] Lai XX Xu RA Yu-Ping L Yang H. Risk of Adverse Events With Bevacizumab Addition to Therapy in Advanced non-Small-Cell Lung Cancer: A Meta-Analysis of Randomized Controlled Trials. Onco Targets Ther (2016) 9:2421–8. doi: 10.2147/OTT.S96156 PMC4844428 27143937

[38] Small HY Montezano AC Rios FJ Savoia C Touyz RM. Hypertension Due to Antiangiogenic Cancer Therapy With Vascular Endothelial Growth Factor Inhibitors: Understanding and Managing a New Syndrome. Can J Cardiol (2014) 30:534–43. doi: 10.1016/j.cjca.2014.02.011 24786444

[39] Middleton G Lapka DV. Bevacizumab (Avastin). Clin J Oncol Nurs (2004) 8:666–9. doi: 10.1188/04.CJON.663-669 15637962

[40] Graham J Muhsin M Kirkpatrick P. Cetuximab. Nat Rev Drug Discov (2004) 3:549–50. doi: 10.1038/nrd1445 15272498

[41] Tyagi P. Recent Results and Ongoing Trials With Panitumumab (ABX-EGF), a Fully Human Anti-Epidermal Growth Factor Receptor Antibody, in Metastatic Colorectal Cancer. Clin Colorectal Cancer (2005) 5:21–3. doi: 10.1016/s1533-0028(11)70161-x 15929802

[42] Poole RM Vaidya A. Ramucirumab: First Global Approval. Drugs (2014) 74:1047–58. doi: 10.1007/s40265-014-0244-2 24916147

[43] Thatcher N Hirsch FR Luft AV Szczesna A Ciuleanu TE Dediu M. Necitumumab Plus Gemcitabine and Cisplatin Versus Gemcitabine and Cisplatin Alone as First-Line Therapy in Patients With Stage IV Squamous non-Small-Cell Lung Cancer (SQUIRE): An Open-Label, Randomised, Controlled Phase 3 Trial. Lancet Oncol (2015) 16:763–74. doi: 10.1016/S1470-2045(15)00021-2 26045340

[44] Olaratumab Approved for Soft-Tissue Sarcoma. Cancer Discov (2016) 6:1297. doi: 10.1158/2159-8290.CD-NB2016-141 27827816

[45] Hartmann JT Haap M Kopp HG Lipp HP. Tyrosine Kinase Inhibitors - a Review on Pharmacology, Metabolism and Side Effects. Curr Drug Metab (2009) 10:470–81. doi: 10.2174/138920009788897975 19689244

[46] Zhao Y Bilal M Raza A Khan MI Mehmood S Hayat U. Tyrosine Kinase Inhibitors and Their Unique Therapeutic Potentialities to Combat Cancer. Int J Biol Macromol (2021) 168:22–37. doi: 10.1016/j.ijbiomac.2020.12.009 33290765

[47] Chaar M Kamta J Ait-Oudhia S. Mechanisms, Monitoring, and Management of Tyrosine Kinase Inhibitors-Associated Cardiovascular Toxicities. Onco Targets Ther (2018) 11:6227–37. doi: 10.2147/OTT.S170138 PMC6163027 30288058

[48] Lipsky A Lamanna N. Managing Toxicities of Bruton Tyrosine Kinase Inhibitors. Hematol Am Soc Hematol Educ Program (2020) 2020:336–45. doi: 10.1182/hematology.2020000118 PMC7727553 33275698

[49] Jiao Q Bi L Ren Y Song S Wang Q Wang YS. Advances in Studies of Tyrosine Kinase Inhibitors and Their Acquired Resistance. Mol Cancer (2018) 17:36. doi: 10.1186/s12943-018-0801-5 29455664 PMC5817861

[50] Alam MT Nagao-Kitamoto H Ohga N Akiyama K Maishi N Kawamoto T. Suprabasin as a Novel Tumor Endothelial Cell Marker. Cancer Sci (2014) 105:1533–40. doi: 10.1111/cas.12549 PMC4317965 25283635

[51] Yamamoto K Ohga N Hida Y Maishi N Kawamoto T Kitayama K. Biglycan is a Specific Marker and an Autocrine Angiogenic Factor of Tumour Endothelial Cells. Br J Cancer (2012) 106:1214–23. doi: 10.1038/bjc.2012.59 PMC3304426 22374465

[52] Rohlenova K Goveia J Garcia-Caballero M Subramanian A Kalucka J Treps L. Single-Cell RNA Sequencing Maps Endothelial Metabolic Plasticity in Pathological Angiogenesis. Cell Metab (2020) 31:862–877 e814. doi: 10.1016/j.cmet.2020.03.009 32268117

[53] Maishi N Kikuchi H Sato M Nagao-Kitamoto H Annan DA Baba S. Development of Immortalized Human Tumor Endothelial Cells From Renal Cancer. Int J Mol Sci (2019) 20:4595. doi: 10.3390/ijms20184595 PMC6770423 31533313

[54] Hida K Maishi N Annan DA Hida Y. Contribution of Tumor Endothelial Cells in Cancer Progression. Int J Mol Sci (2018) 19:1272. doi: 10.3390/ijms19051272 PMC5983794 29695087

[55] Eelen G de Zeeuw P Simons M Carmeliet P. Endothelial Cell Metabolism in Normal and Diseased Vasculature. Circ Res (2015) 116:1231–44. doi: 10.1161/CIRCRESAHA.116.302855 PMC4380230 25814684

[56] Chen WZ Jiang JX Yu XY Xia WJ Yu PX Wang K. Endothelial Cells in Colorectal Cancer. World J Gastrointest Oncol (2019) 11:946–56. doi: 10.4251/wjgo.v11.i11.946 PMC6883186 31798776

[57] Morikawa S Baluk P Kaidoh T Haskell A Jain RK McDonald DM. Abnormalities in Pericytes on Blood Vessels and Endothelial Sprouts in Tumors. Am J Pathol (2002) 160:985–1000. doi: 10.1016/S0002-9440(10)64920-6 11891196 PMC1867175

[58] Hida K Hida Y Amin DN Flint AF Panigrahy D Morton CC. Tumor-Associated Endothelial Cells With Cytogenetic Abnormalities. Cancer Res (2004) 64:8249–55. doi: 10.1158/0008-5472.CAN-04-1567 15548691

[59] Akino T Hida K Hida Y Tsuchiya K Freedman D Muraki C. Cytogenetic Abnormalities of Tumor-Associated Endothelial Cells in Human Malignant Tumors. Am J Pathol (2009) 175:2657–67. doi: 10.2353/ajpath.2009.090202 PMC2789618 19875502

[60] Streubel B Chott A Huber D Exner M Jager U Wagner O. Lymphoma-Specific Genetic Aberrations in Microvascular Endothelial Cells in B-Cell Lymphomas. N Engl J Med (2004) 351:250–9. doi: 10.1056/NEJMoa033153 15254283

[61] Chai ZT Zhang XP Ao JY Zhu XD Wu MC Lau WY. AXL Overexpression in Tumor-Derived Endothelial Cells Promotes Vessel Metastasis in Patients With Hepatocellular Carcinoma. Front Oncol (2021) 11:650963. doi: 10.3389/fonc.2021.650963 34123800 PMC8191462

[62] Mesri M Birse C Heidbrink J McKinnon K Brand E Bermingham CL. Identification and Characterization of Angiogenesis Targets Through Proteomic Profiling of Endothelial Cells in Human Cancer Tissues. PloS One (2013) 8:e78885. doi: 10.1371/journal.pone.0078885 24236063 PMC3827283

[63] Bussolati B Deambrosis I Russo S Deregibus MC Camussi G. Altered Angiogenesis and Survival in Human Tumor-Derived Endothelial Cells. FASEB J (2003) 17:1159–61. doi: 10.1096/fj.02-0557fje 12709414

[64] Maishi N Annan DA Kikuchi H Hida Y Hida K. Tumor Endothelial Heterogeneity in Cancer Progression. Cancers (Basel) (2019) 11:1511. doi: 10.3390/cancers11101511 PMC6826555 31600937

[65] Ricci-Vitiani L Pallini R Biffoni M Todaro M Invernici G Cenci T. Tumour Vascularization *via* Endothelial Differentiation of Glioblastoma Stem-Like Cells. Nature (2010) 468:824–8. doi: 10.1038/nature09557 21102434

[66] Pezzolo A Parodi F Corrias MV Cinti R Gambini C Pistoia V. Tumor Origin of Endothelial Cells in Human Neuroblastoma. J Clin Oncol (2007) 25:376–83. doi: 10.1200/JCO.2006.09.0696 17264333

[67] Marcola M Rodrigues CE. Endothelial Progenitor Cells in Tumor Angiogenesis: Another Brick in the Wall. Stem Cells Int (2015) 2015:832649. doi: 10.1155/2015/832649 26000021 PMC4427119

[68] Torii C Maishi N Kawamoto T Morimoto M Akiyama K Yoshioka Y. Mirna-1246 in Extracellular Vesicles Secreted From Metastatic Tumor Induces Drug Resistance in Tumor Endothelial Cells. Sci Rep (2021) 11:13502. doi: 10.1038/s41598-021-92879-5 34226586 PMC8257582

[69] Cheng HW Chen YF Wong JM Weng CW Chen HY Yu SL. Cancer Cells Increase Endothelial Cell Tube Formation and Survival by Activating the PI3K/Akt Signalling Pathway. J Exp Clin Cancer Res (2017) 36:27. doi: 10.1186/s13046-017-0495-3 28173828 PMC5296960

[70] Ciesielski O Biesiekierska M Panthu B Vialichka V Pirola L Balcerczyk A. The Epigenetic Profile of Tumor Endothelial Cells. Effects of Combined Therapy With Antiangiogenic and Epigenetic Drugs on Cancer Progression. Int J Mol Sci (2020) 21:2606. doi: 10.3390/ijms21072606 PMC7177242 32283668

[71] Brooks MD Burness ML Wicha MS. Therapeutic Implications of Cellular Heterogeneity and Plasticity in Breast Cancer. Cell Stem Cell (2015) 17:260–71. doi: 10.1016/j.stem.2015.08.014 PMC4560840 26340526

[72] Sotgia F Fiorillo M Lisanti MP. Hallmarks of the Cancer Cell of Origin: Comparisons With “Energetic” Cancer Stem Cells (E-Cscs). Aging (Albany NY) (2019) 11:1065–8. doi: 10.18632/aging.101822 PMC6382415 30760648

[73] Zhao H Yan C Hu Y Mu L Liu S Huang K. Differentiated Cancer Cell-Originated Lactate Promotes the Self-Renewal of Cancer Stem Cells in Patient-Derived Colorectal Cancer Organoids. Cancer Lett (2020) 493:236–44. doi: 10.1016/j.canlet.2020.08.044 32898601

[74] O’Brien CA Kreso A Ryan P Hermans KG Gibson L Wang Y. ID1 and ID3 Regulate the Self-Renewal Capacity of Human Colon Cancer-Initiating Cells Through P21. Cancer Cell (2012) 21:777–92. doi: 10.1016/j.ccr.2012.04.036 22698403

[75] Kumar D Kumar S Gorain M Tomar D Patil HS Radharani NNV. Notch1-MAPK Signaling Axis Regulates CD133(+) Cancer Stem Cell-Mediated Melanoma Growth and Angiogenesis. J Invest Dermatol (2016) 136:2462–74. doi: 10.1016/j.jid.2016.07.024 27476721

[76] Hayakawa Y Ariyama H Stancikova J Sakitani K Asfaha S Renz BW. Mist1 Expressing Gastric Stem Cells Maintain the Normal and Neoplastic Gastric Epithelium and Are Supported by a Perivascular Stem Cell Niche. Cancer Cell (2015) 28:800–14. doi: 10.1016/j.ccell.2015.10.003 PMC4684751 26585400

[77] Feng C Yang X Sun J Luo Q Song G. [Effect of Conditioned Medium From Endothelial Cells on Cancer Stem Cell Phenotype of Hepatoma Cells]. Sheng Wu Yi Xue Gong Cheng Xue Za Zhi (2015) 32:1061–6. 26964312

[78] Lu J Ye X Fan F Xia L Bhattacharya R Bellister S. Endothelial Cells Promote the Colorectal Cancer Stem Cell Phenotype Through a Soluble Form of Jagged-1. Cancer Cell (2013) 23:171–85. doi: 10.1016/j.ccr.2012.12.021 PMC3574187 23375636

[79] Wang Y Wang Y Chen H Liang Q. Endothelial Cells Promote Formation of Medulloblastoma Stem-Like Cells *via* Notch Pathway Activation. J Mol Neurosci (2017) 63:152–8. doi: 10.1007/s12031-017-0965-2 28856557

[80] Mendonca L Trindade A Carvalho C Correia J Badenes M Gigante J. Metastasis is Impaired by Endothelial-Specific Dll4 Loss-of-Function Through Inhibition of Epithelial-to-Mesenchymal Transition and Reduction of Cancer Stem Cells and Circulating Tumor Cells. Clin Exp Metastasis (2019) 36:365–80. doi: 10.1007/s10585-019-09973-2 31119445

[81] Kong DH Kim MR Jang JH Na HJ Lee S. A Review of Anti-Angiogenic Targets for Monoclonal Antibody Cancer Therapy. Int J Mol Sci (2017) 18:1786. doi: 10.3390/ijms18081786 PMC5578174 28817103

[82] Sun L Pan J Yu L Liu H Shu X Sun L. Tumor Endothelial Cells Promote Metastasis and Cancer Stem Cell-Like Phenotype Through Elevated Epiregulin in Esophageal Cancer. Am J Cancer Res (2016) 6:2277–88. PMC5088291 27822417

[83] Thomann S Weiler SME Marquard S Rose F Ball CR Toth M. YAP Orchestrates Heterotypic Endothelial Cell Communication *via* HGF/C-MET Signaling in Liver Tumorigenesis. Cancer Res (2020) 80:5502–14. doi: 10.1158/0008-5472.CAN-20-0242 33087321

[84] Ritchey L Ha T Otsuka A Kabashima K Wang D Wang Y. DLC1 Deficiency and YAP Signaling Drive Endothelial Cell Contact Inhibition of Growth and Tumorigenesis. Oncogene (2019) 38:7046–59. doi: 10.1038/s41388-019-0944-x PMC8276116 31409902

[85] Ye C Hu Y Wang J Liu D Du J. Mono (2-Ethylhexyl) Phthalate (MEHP) Triggers the Proliferation of Hemangioma-Derived Endothelial Cells *via* YAP Signals. Chem Biol Interact (2019) 311:108773. doi: 10.1016/j.cbi.2019.108773 31351048

[86] Hooglugt A van der Stoel MM Boon RA Huveneers S. Endothelial YAP/TAZ Signaling in Angiogenesis and Tumor Vasculature. Front Oncol (2020) 10:612802. doi: 10.3389/fonc.2020.612802 33614496 PMC7890025

[87] Ciszewski WM Sobierajska K Wawro ME Klopocka W Chefczynska N Muzyczuk A. the ILK-MMP9-MRTF Axis is Crucial for Endmt Differentiation of Endothelial Cells in a Tumor Microenvironment. Biochim Biophys Acta Mol Cell Res (2017) 1864:2283–96. doi: 10.1016/j.bbamcr.2017.09.004 28893556

[88] Krizbai IA Gasparics A Nagyoszi P Fazakas C Molnar J Wilhelm I. Endothelial-Mesenchymal Transition of Brain Endothelial Cells: Possible Role During Metastatic Extravasation. PloS One (2015) 10:e0119655. doi: 10.1371/journal.pone.0119655 25742314 PMC4350839

[89] Fan CS Chen WS Chen LL Chen CC Hsu YT Chua KV. Osteopontin-Integrin Engagement Induces HIF-1alpha-TCF12-Mediated Endothelial-Mesenchymal Transition to Exacerbate Colorectal Cancer. Oncotarget (2018) 9:4998–5015. doi: 10.18632/oncotarget.23578 29435158 PMC5797029

[90] Anderberg C Cunha SI Zhai Z Cortez E Pardali E Johnson JR. Deficiency for Endoglin in Tumor Vasculature Weakens the Endothelial Barrier to Metastatic Dissemination. J Exp Med (2013) 210:563–79. doi: 10.1084/jem.20120662 PMC3600899 23401487

[91] Kim SH Song Y Seo HR. GSK-3beta Regulates the Endothelial-to-Mesenchymal Transition *via* Reciprocal Crosstalk Between NSCLC Cells and Huvecs in Multicellular Tumor Spheroid Models. J Exp Clin Cancer Res (2019) 38:46. doi: 10.1186/s13046-019-1050-1 30709379 PMC6359813

[92] Laferriere J Houle F Taher MM Valerie K Huot J. Transendothelial Migration of Colon Carcinoma Cells Requires Expression of E-Selectin by Endothelial Cells and Activation of Stress-Activated Protein Kinase-2 (SAPK2/P38) in the Tumor Cells. J Biol Chem (2001) 276:33762–72. doi: 10.1074/jbc.M008564200 11448946

[93] Tremblay PL Auger FA Huot J. Regulation of Transendothelial Migration of Colon Cancer Cells by E-Selectin-Mediated Activation of P38 and ERK MAP Kinases. Oncogene (2006) 25:6563–73. doi: 10.1038/sj.onc.1209664 16715142

[94] Ghislin S Obino D Middendorp S Boggetto N Alcaide-Loridan C Deshayes F. LFA-1 and ICAM-1 Expression Induced During Melanoma-Endothelial Cell Co-Culture Favors the Transendothelial Migration of Melanoma Cell Lines In Vitro. BMC Cancer (2012) 12:455. doi: 10.1186/1471-2407-12-455 23039186 PMC3495854

[95] Sahni A Arevalo MT Sahni SK Simpson-Haidaris PJ. the VE-Cadherin Binding Domain of Fibrinogen Induces Endothelial Barrier Permeability and Enhances Transendothelial Migration of Malignant Breast Epithelial Cells. Int J Cancer (2009) 125:577–84. doi: 10.1002/ijc.24340 19358279

[96] Arlt MJ Novak-Hofer I Gast D Gschwend V Moldenhauer G Grunberg J. Efficient Inhibition of Intra-Peritoneal Tumor Growth and Dissemination of Human Ovarian Carcinoma Cells in Nude Mice by Anti-L1-Cell Adhesion Molecule Monoclonal Antibody Treatment. Cancer Res (2006) 66:936–43. doi: 10.1158/0008-5472.CAN-05-1818 16424028

[97] Huang L Perrault C Coelho-Martins J Hu C Dulong C Varna M. Induction of Acquired Drug Resistance in Endothelial Cells and Its Involvement in Anticancer Therapy. J Hematol Oncol (2013) 6:49. doi: 10.1186/1756-8722-6-49 23837843 PMC3717049

[98] Yu Z Mouillesseaux KP Kushner EJ Bautch VL. Tumor-Derived Factors and Reduced P53 Promote Endothelial Cell Centrosome Over-Duplication. PloS One (2016) 11:e0168334. doi: 10.1371/journal.pone.0168334 27977771 PMC5158050

[99] Xiong YQ Sun HC Zhang W Zhu XD Zhuang PY Zhang JB. Human Hepatocellular Carcinoma Tumor-Derived Endothelial Cells Manifest Increased Angiogenesis Capability and Drug Resistance Compared With Normal Endothelial Cells. Clin Cancer Res (2009) 15:4838–46. doi: 10.1158/1078-0432.CCR-08-2780 19638466

[100] Ohga N Ishikawa S Maishi N Akiyama K Hida Y Kawamoto T. Heterogeneity of Tumor Endothelial Cells: Comparison Between Tumor Endothelial Cells Isolated From High- and Low-Metastatic Tumors. Am J Pathol (2012) 180:1294–307. doi: 10.1016/j.ajpath.2011.11.035 22245217

[101] Huang L Hu C Di Benedetto M Varin R Liu J Wang L. Induction of Multiple Drug Resistance in HMEC-1 Endothelial Cells After Long-Term Exposure to Sunitinib. Onco Targets Ther (2014) 7:2249–55. doi: 10.2147/OTT.S67251 PMC4262216 25587220

[102] Akiyama K Ohga N Hida Y Kawamoto T Sadamoto Y Ishikawa S. Tumor Endothelial Cells Acquire Drug Resistance by MDR1 Up-Regulation *via* VEGF Signaling in Tumor Microenvironment. Am J Pathol (2012) 180:1283–93. doi: 10.1016/j.ajpath.2011.11.029 22245726

[103] Nielsen N Lindemann O Schwab A. TRP Channels and STIM/ORAI Proteins: Sensors and Effectors of Cancer and Stroma Cell Migration. Br J Pharmacol (2014) 171:5524–40. doi: 10.1111/bph.12721 PMC4290700 24724725

[104] Moccia F. Endothelial Ca(2+) Signaling and the Resistance to Anticancer Treatments: Partners in Crime. Int J Mol Sci (2018) 19:217. doi: 10.3390/ijms19010217 PMC5796166 29324706

[105] Ebos JM Lee CR Christensen JG Mutsaers AJ Kerbel RS. Multiple Circulating Proangiogenic Factors Induced by Sunitinib Malate Are Tumor-Independent and Correlate With Antitumor Efficacy. Proc Natl Acad Sci USA (2007) 104:17069–74. doi: 10.1073/pnas.0708148104 PMC2040401 17942672

[106] Song Y Tang C Yin C. Combination Antitumor Immunotherapy With VEGF and PIGF SiRNA *via* Systemic Delivery of Multi-Functionalized Nanoparticles to Tumor-Associated Macrophages and Breast Cancer Cells. Biomaterials (2018) 185:117–32. doi: 10.1016/j.biomaterials.2018.09.017 30241030

[107] Ohashi K Wang Z Yang YM Billet S Tu W Pimienta M. NOD-Like Receptor C4 Inflammasome Regulates the Growth of Colon Cancer Liver Metastasis in NAFLD. Hepatology (2019) 70:1582–99. doi: 10.1002/hep.30693 PMC6819206 31044438

[108] Wang D Wang X Si M Yang J Sun S Wu H. Exosome-Encapsulated Mirnas Contribute to CXCL12/CXCR4-Induced Liver Metastasis of Colorectal Cancer by Enhancing M2 Polarization of Macrophages. Cancer Lett (2020) 474:36–52. doi: 10.1016/j.canlet.2020.01.005 31931030

[109] Huang JK Ma L Song WH Lu BY Huang YB Dong HM. Lncrna-MALAT1 Promotes Angiogenesis of Thyroid Cancer by Modulating Tumor-Associated Macrophage FGF2 Protein Secretion. J Cell Biochem (2017) 118:4821–30. doi: 10.1002/jcb.26153 28543663

[110] Lv J Liu C Chen FK Feng ZP Jia L Liu PJ. M2like Tumourassociated Macrophagesecreted IGF Promotes Thyroid Cancer Stemness and Metastasis by Activating the PI3K/AKT/Mtor Pathway. Mol Med Rep (2021) 24:604. doi: 10.3892/mmr.2021.12249 34184083 PMC8258465

[111] Liu Q Song J Pan Y Shi D Yang C Wang S. Wnt5a/Camkii/ERK/CCL2 Axis is Required for Tumor-Associated Macrophages to Promote Colorectal Cancer Progression. Int J Biol Sci (2020) 16:1023–34. doi: 10.7150/ijbs.40535 PMC7053330 32140070

[112] Lee S Lee E Ko E Ham M Lee HM Kim ES. Tumor-Associated Macrophages Secrete CCL2 and Induce the Invasive Phenotype of Human Breast Epithelial Cells Through Upregulation of ERO1-Alpha and MMP-9. Cancer Lett (2018) 437:25–34. doi: 10.1016/j.canlet.2018.08.025 30165193

[113] Rolny C Mazzone M Tugues S Laoui D Johansson I Coulon C. HRG Inhibits Tumor Growth and Metastasis by Inducing Macrophage Polarization and Vessel Normalization Through Downregulation of Plgf. Cancer Cell (2011) 19:31–44. doi: 10.1016/j.ccr.2010.11.009 21215706

[114] Yamaguchi T Fushida S Yamamoto Y Tsukada T Kinoshita J Oyama K. Tumor-Associated Macrophages of the M2 Phenotype Contribute to Progression in Gastric Cancer With Peritoneal Dissemination. Gastric Cancer (2016) 19:1052–65. doi: 10.1007/s10120-015-0579-8 PMC5034006 26621525

[115] Tauchi Y Tanaka H Kumamoto K Tokumoto M Sakimura C Sakurai K. Tumor-Associated Macrophages Induce Capillary Morphogenesis of Lymphatic Endothelial Cells Derived From Human Gastric Cancer. Cancer Sci (2016) 107:1101–9. doi: 10.1111/cas.12977 PMC4982583 27227358

[116] Lin L Chen YS Yao YD Chen JQ Chen JN Huang SY. CCL18 From Tumor-Associated Macrophages Promotes Angiogenesis in Breast Cancer. Oncotarget (2015) 6:34758–73. doi: 10.18632/oncotarget.5325 PMC4741488 26416449

[117] Teuwen LA De Rooij L Cuypers A Rohlenova K Dumas SJ Garcia-Caballero M. Tumor Vessel Co-Option Probed by Single-Cell Analysis. Cell Rep (2021) 35:109253. doi: 10.1016/j.celrep.2021.109253 34133923

[118] Mazzieri R Pucci F Moi D Zonari E Ranghetti A Berti A. Targeting the ANG2/TIE2 Axis Inhibits Tumor Growth and Metastasis by Impairing Angiogenesis and Disabling Rebounds of Proangiogenic Myeloid Cells. Cancer Cell (2011) 19:512–26. doi: 10.1016/j.ccr.2011.02.005 21481792

[119] Kang JH Woo JK Jang YS Oh SH. Radiation Potentiates Monocyte Infiltration Into Tumors by Ninjurin1 Expression in Endothelial Cells. Cells (2020) 9:1086. doi: 10.3390/cells9051086 PMC7291157 32353975

[120] Yin M Zhou HJ Zhang J Lin C Li H Li X. ASK1-Dependent Endothelial Cell Activation is Critical in Ovarian Cancer Growth and Metastasis. JCI Insight (2017) 2:e91828. doi: 10.1172/jci.insight.91828 PMC5621912 28931753

[121] He H Xu J Warren CM Duan D Li X Wu L. Endothelial Cells Provide an Instructive Niche for the Differentiation and Functional Polarization of M2-Like Macrophages. Blood (2012) 120:3152–62. doi: 10.1182/blood-2012-04-422758 PMC3471522 22919031

[122] Zhou J Zhang A Fan L. HSPA12B Secreted by Tumor-Associated Endothelial Cells Might Induce M2 Polarization of Macrophages *via* Activating PI3K/Akt/Mtor Signaling. Onco Targets Ther (2020) 13:9103–11. doi: 10.2147/OTT.S254985 PMC7494226 32982299

[123] Kim K Sohn YJ Lee R Yoo HJ Kang JY Choi N. Cancer-Associated Fibroblasts Differentiated by Exosomes Isolated From Cancer Cells Promote Cancer Cell Invasion. Int J Mol Sci (2020) 21:8153. doi: 10.3390/ijms21218153 PMC7662577 33142759

[124] Sobierajska K Ciszewski WM Sacewicz-Hofman I Niewiarowska J. Endothelial Cells in the Tumor Microenvironment. Adv Exp Med Biol (2020) 1234:71–86. doi: 10.1007/978-3-030-37184-5_6 32040856

[125] van Meeteren LA ten Dijke P. Regulation of Endothelial Cell Plasticity by TGF-Beta. Cell Tissue Res (2012) 347:177–86. doi: 10.1007/s00441-011-1222-6 PMC3250609 21866313

[126] Sanchez-Duffhues G Orlova V Ten Dijke P. in Brief: Endothelial-to-Mesenchymal Transition. J Pathol (2016) 238:378–80. doi: 10.1002/path.4653 26446982

[127] Yeon JH Jeong HE Seo H Cho S Kim K Na D. Cancer-Derived Exosomes Trigger Endothelial to Mesenchymal Transition Followed by the Induction of Cancer-Associated Fibroblasts. Acta Biomater (2018) 76:146–53. doi: 10.1016/j.actbio.2018.07.001 30078422

[128] Fukumura D Xavier R Sugiura T Chen Y Park EC Lu N. Tumor Induction of VEGF Promoter Activity in Stromal Cells. Cell (1998) 94:715–25. doi: 10.1016/s0092-8674(00)81731-6 9753319

[129] Pietras K Pahler J Bergers G Hanahan D. Functions of Paracrine PDGF Signaling in the Proangiogenic Tumor Stroma Revealed by Pharmacological Targeting. PloS Med (2008) 5:e19. doi: 10.1371/journal.pmed.0050019 18232728 PMC2214790

[130] Orimo A Gupta PB Sgroi DC Arenzana-Seisdedos F Delaunay T Naeem R. Stromal Fibroblasts Present in Invasive Human Breast Carcinomas Promote Tumor Growth and Angiogenesis Through Elevated SDF-1/CXCL12 Secretion. Cell (2005) 121:335–48. doi: 10.1016/j.cell.2005.02.034 15882617

[131] Crawford Y Kasman I Yu L Zhong C Wu X Modrusan Z. PDGF-C Mediates the Angiogenic and Tumorigenic Properties of Fibroblasts Associated With Tumors Refractory to Anti-VEGF Treatment. Cancer Cell (2009) 15:21–34. doi: 10.1016/j.ccr.2008.12.004 19111878

[132] Leung CS Yeung TL Yip KP Wong KK Ho SY Mangala LS. Cancer-Associated Fibroblasts Regulate Endothelial Adhesion Protein LPP to Promote Ovarian Cancer Chemoresistance. J Clin Invest (2018) 128:589–606. doi: 10.1172/JCI95200 29251630 PMC5785271

[133] Khaled YS Ammori BJ Elkord E. Myeloid-Derived Suppressor Cells in Cancer: Recent Progress and Prospects. Immunol Cell Biol (2013) 91:493–502. doi: 10.1038/icb.2013.29 23797066

[134] Zheng W Zhu Y Chen X Zhao J. CD73 Expression in Myeloid-Derived Suppressor Cells is Correlated With Clinical Stages in Head and Neck Squamous Cell Carcinomas. Ann Transl Med (2021) 9:1148. doi: 10.21037/atm-21-2589 34430589 PMC8350661

[135] Kumar V Cheng P Condamine T Mony S Languino LR McCaffrey JC. CD45 Phosphatase Inhibits STAT3 Transcription Factor Activity in Myeloid Cells and Promotes Tumor-Associated Macrophage Differentiation. Immunity (2016) 44:303–15. doi: 10.1016/j.immuni.2016.01.014 PMC4759655 26885857

[136] Kumar V Patel S Tcyganov E Gabrilovich DI. the Nature of Myeloid-Derived Suppressor Cells in the Tumor Microenvironment. Trends Immunol (2016) 37:208–20. doi: 10.1016/j.it.2016.01.004 PMC4775398 26858199

[137] Kumar S Wilkes DW Samuel N Blanco MA Nayak A Alicea-Torres K. Deltanp63-Driven Recruitment of Myeloid-Derived Suppressor Cells Promotes Metastasis in Triple-Negative Breast Cancer. J Clin Invest (2018) 128:5095–109. doi: 10.1172/JCI99673 PMC6205409 30295647

[138] Taki M Abiko K Baba T Hamanishi J Yamaguchi K Murakami R. Snail Promotes Ovarian Cancer Progression by Recruiting Myeloid-Derived Suppressor Cells *via* CXCR2 Ligand Upregulation. Nat Commun (2018) 9:1685. doi: 10.1038/s41467-018-03966-7 29703902 PMC5923228

[139] Pellegrino A Ria R Di Pietro G Cirulli T Surico G Pennisi A. Bone Marrow Endothelial Cells in Multiple Myeloma Secrete CXC-Chemokines That Mediate Interactions With Plasma Cells. Br J Haematol (2005) 129:248–56. doi: 10.1111/j.1365-2141.2005.05443.x 15813853

[140] Salazar N Zabel BA. Support of Tumor Endothelial Cells by Chemokine Receptors. Front Immunol (2019) 10:147. doi: 10.3389/fimmu.2019.00147 30800123 PMC6375834

[141] Li BH Garstka MA Li ZF. Chemokines and Their Receptors Promoting the Recruitment of Myeloid-Derived Suppressor Cells Into the Tumor. Mol Immunol (2020) 117:201–15. doi: 10.1016/j.molimm.2019.11.014 31835202

[142] Oliveira AC Fu C Lu Y Williams MA Pi L Brantly ML. Chemokine Signaling Axis Between Endothelial and Myeloid Cells Regulates Development of Pulmonary Hypertension Associated With Pulmonary Fibrosis and Hypoxia. Am J Physiol Lung Cell Mol Physiol (2019) 317:L434–44. doi: 10.1152/ajplung.00156.2019 PMC6842914 31364370

[143] Yin J Shen Y Yu A Liu C Yao J Gong W. The Proangiogenic Role of Polymorphonuclear Myeloid-Derived Suppressor Cells in Mice Infected With Echinococcus Granulosus. Biosci Trends (2018) 12:338–41. doi: 10.5582/bst.2018.01105 30012917

[144] Wang W Zuo R Long H Wang Y Zhang Y Sun C. Advances in the Masquelet Technique: Myeloid-Derived Suppressor Cells Promote Angiogenesis in PMMA-Induced Membranes. Acta Biomater (2020) 108:223–36. doi: 10.1016/j.actbio.2020.03.010 32165192

[145] Sack KD Teran M Nugent MA. Extracellular Matrix Stiffness Controls VEGF Signaling and Processing in Endothelial Cells. J Cell Physiol (2016) 231:2026–39. doi: 10.1002/jcp.25312 26773314

[146] Bao M Chen Y Liu JT Bao H Wang WB Qi YX. Extracellular Matrix Stiffness Controls VEGF165 Secretion and Neuroblastoma Angiogenesis *via* the YAP/RUNX2/SRSF1 Axis. Angiogenesis (2021). doi: 10.1007/s10456-021-09804-7 34170441

[147] Davidson CD Jayco DKP Wang WY Shikanov A Baker BM. Fiber Crimp Confers Matrix Mechanical Nonlinearity, Regulates Endothelial Cell Mechanosensing, and Promotes Microvascular Network Formation. J Biomech Eng (2020) 142:111009. doi: 10.1115/1.4048191 32839824 PMC7580766

[148] Chetty C Lakka SS Bhoopathi P Rao JS. MMP-2 Alters VEGF Expression *via* Alphavbeta3 Integrin-Mediated PI3K/AKT Signaling in A549 Lung Cancer Cells. Int J Cancer (2010) 127:1081–95. doi: 10.1002/ijc.25134 PMC2891576 20027628

[149] Bayless KJ Salazar R Davis GE. RGD-Dependent Vacuolation and Lumen Formation Observed During Endothelial Cell Morphogenesis in Three-Dimensional Fibrin Matrices Involves the Alpha(V)Beta(3) and Alpha(5)Beta(1) Integrins. Am J Pathol (2000) 156:1673–83. doi: 10.1016/s0002-9440(10)65038-9 PMC1876924 10793078

[150] Peyri N Berard M Fauvel-Lafeve F Trochon V Arbeille B Lu H. Breast Tumor Cells Transendothelial Migration Induces Endothelial Cell Anoikis Through Extracellular Matrix Degradation. Anticancer Res (2009) 29:2347–55. 19528501

[151] Krzyzanowska-Golab D Lemanska-Perek A Katnik-Prastowska I. Fibronectin as an Active Component of the Extracellular Matrix. Postepy Hig Med Dosw (Online) (2007) 61:655–63. 17989620

[152] Yoshimatsu Y Wakabayashi I Kimuro S Takahashi N Takahashi K Kobayashi M. TNF-Alpha Enhances TGF-Beta-Induced Endothelial-to-Mesenchymal Transition *via* TGF-Beta Signal Augmentation. Cancer Sci (2020) 111:2385–99. doi: 10.1111/cas.14455 PMC7385392 32385953

